# Chiral Stationary Phases for Liquid Chromatography: Recent Developments

**DOI:** 10.3390/molecules24050865

**Published:** 2019-02-28

**Authors:** Joana Teixeira, Maria Elizabeth Tiritan, Madalena M. M. Pinto, Carla Fernandes

**Affiliations:** 1 Laboratório de Química Orgânica e Farmacêutica, Departamento de Ciências Químicas, Faculdade de Farmácia, Universidade do Porto, Rua de Jorge Viterbo Ferreira, 228, 4050-313 Porto, Portugal; jbteixeira@live.com.pt (J.T.); elizabeth.tiritan@iucs.cespu.pt (M.E.T.); madalena@ff.up.pt (M.M.M.P.); 2 Interdisciplinary Centre of Marine and Environmental Research (CIIMAR), Edifício do Terminal de Cruzeiros do Porto de Leixões, Av. General Norton de Matos s/n, 4050-208 Matosinhos, Portugal; 3 Cooperativa de Ensino Superior, Politécnico e Universitário (CESPU), Instituto de Investigação e Formação Avançada em Ciências e Tecnologias da Saúde (IINFACTS), Rua Central de Gandra, 1317, 4585-116 Gandra PRD, Portugal

**Keywords:** liquid chromatography, enantioseparation, chiral stationary phase, chiral selector, chromatographic support

## Abstract

The planning and development of new chiral stationary phases (CSPs) for liquid chromatography (LC) are considered as continuous and evolutionary issues since the introduction of the first CSP in 1938. The main objectives of the development strategies were to attempt the improvement of the chromatographic enantioresolution performance of the CSPs as well as enlarge their versatility and range of applications. Additionally, the transition to ultra-high-performance LC were underscored. The most recent strategies have comprised the introduction of new chiral selectors, the use of new materials as chromatographic supports or the reduction of its particle size, and the application of different synthetic approaches for preparation of CSPs. This review gathered the most recent developments associated to the different types of CSPs providing an overview of the relevant advances that are arising on LC.

## 1. Introduction

Now more than ever, analytical and preparative enantiomeric separations play a crucial role in industry and academic research [1]. There are a wide variety of methods to achieve and analyze enantiomerically pure compounds, including liquid chromatography (LC) [2,3], supercritical fluid chromatography [4,5,6], diastereomeric crystallization [7,8], membranes [9,10], asymmetric catalysis [11], simulated moving bed [12,13], dynamic and enzyme-mediated kinetic resolution [14,15], among others. 

LC using chiral stationary phases (CSPs) proved to be an essential tool with a wide range of applications, including preparative separation of enantiomers of diverse analytes [16,17], determination of enantiomeric composition [18,19], monitorization of asymmetric reactions [20,21], analysis of the stereochemistry of natural compounds [22,23], pharmacokinetic [24,25], forensic [26,27,28], environmental [29,30,31], and enantioselective studies [32,33], among others. 

The development of CSPs for LC combined with the improvement of chromatography instrumentation revolutionized the enantioseparation approaches. LC using CSPs has demonstrated to be extremely useful, accurate, versatile, and it has been a widely used technique in diverse fields and applications, emphasizing, for example, the enantioseparation of underivatized amino acids [34,35], diverse classes of pharmaceuticals [36,37,38,39,40], atropisomers [41], as well as the study of intermolecular interactions between biomolecules and drugs [42], among others.

Over the last decades, several types of CSPs have been developed [43,44,45,46] and, among them, more than a hundred are currently commercially available [39]. These comprise Pirkle-type, ligand-exchange-type, molecularly-imprinted, and based on macrocyclic antibiotics, proteins, polysaccharides, cyclodextrins, crown ethers, cyclofructans, synthetic polymers, among others [43,44,45,46]. Nevertheless, although many different types of CSPs are described, the development of new CSPs continues to be a field of research with great importance.

## 2. Chiral Stationary Phases: Recent Developments

Since the first description of a CSP, in 1938, by Henderson and Rule [47], and to follow the constant challenges on different areas as well as the advances in chromatographic instrumentation, the development of new CSPs for LC has been a continuous and evolutionary subject. 

**Figure 1:**
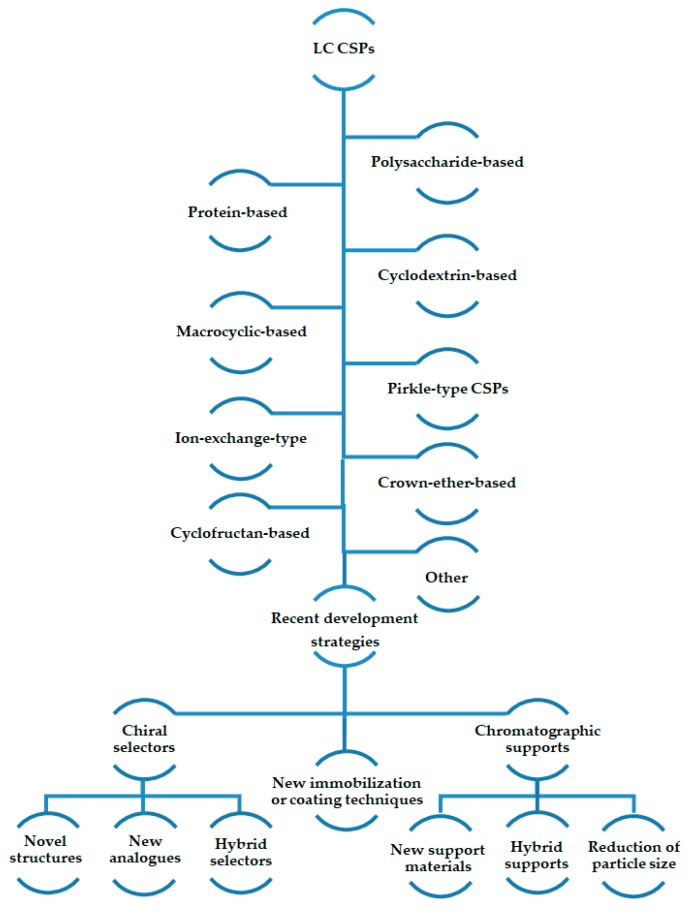
Summary of recent strategies for development of new chiral stationary phases (CSPs) for liquid chromatography (LC).

In this review, for each type of CSPs, the most recent CSPs were presented describing the strategies used for their development (Figure 1).

Herein, only the CSPs that were not reported in previous fundamental reviews will be presented, highlighting the main advantages of the strategy used for their preparation, the best chromatographic results and the objectives to be achieved.

### 2.1. Polysaccharide-Based CSPs

The first application of a polysaccharide as a chromatographic chiral selector was described by Hessen and Hagel, in 1976 [48]. Since then, different polysaccharides were extensively used as CSPs due to their high enantioselectivity properties after derivatization [49]. Nevertheless, amylose and cellulose are the main polysaccharides used to obtain CSPs [50], followed by chitosan and chitin [51]. The chiral recognition ability of polysaccharide derivatives is dependent on diverse structural features, including sugar units, stereogenic centres of the glucopyranose units, type of the linkage and its position, as well as the adjacent polymer chains [49]. The helical twist of the polymer backbone is also essential for enantioselectivity [43]. 

Polysaccharide derivatives as efficient chiral selectors can include phenyl, alkyl or benzylcarbamates, esters, benzoate, or aryl or cycloalkyl groups [52]. Benzoate or phenylcarbamate moieties may comprise methyl, methoxy, among other groups, and/or chlorine substituents in the aromatic ring [52], affording different solubility and chiral recognition ability [52]. Moreover, the position of the substituents in the aromatic ring influences the enantioseparation performance of the chiral selector [49]. 

Polysaccharide derivatives can be coated onto a chromatographic support, as silica or derivatives, by an adsorption process [53,54] allowing a larger surface area [55] and high efficiency [43]. CSPs comprising coated polysaccharide derivatives can operate in normal phase, polar organic and reversed-phase elution mode; however, they have restrictions due to the non-compatibility with “non-standard” solvents, such as dichloromethane, chloroform, toluene, or acetone [43]. The use of those solvents in the mobile phase may cause the dissolution of the adsorbed polymer and, consequently, removal of the selector from the chromatographic column [43]. Immobilized polysaccharides emerged as a reliable alternative allowing the use of a broader selection of solvents as mobile phases [56,57,58,59,60]. Different procedures can be used for covalently bonded the polysaccharide derivatives to the chromatographic support, such as a polymerization reaction and photoinduced and enzymatic polymerization [56]. Nevertheless, despite the solvent versatility, in general, the potential of chiral recognition of immobilized polysaccharide-based CSPs is lower than the coated due to modification of stereospecific conformation that can occur during the immobilization process [56,57]. 

This type of CSPs is recognized as being the most successful and widely applied for both analytical [61,62,63,64,65,66,67,68,69] and preparative enantioseparations [17,70,71,72,73,74,75,76], being responsible for about 99% of reported chiral separations [50]. Among the developed polysaccharide-based CSPs, the 3,5-dimethylphenyl *tris*-phenylcarbamates of amylose and cellulose proved to have the best enantiorecognition performance [77,78,79,80]. In our group, this type of CSPs has proved to be effective for analytical as well as preparative applications [26,81,82,83,84]. 

The chiral recognition mechanisms concerning these CSPs are not yet completely understood. In an attempt to improve the knowledge related with structural features associated with the chiral recognition mechanisms and their chromatographic behavior at a molecular level, several studies concerning to docking, spectroscopy, molecular modelling, and quantum chemical calculations were recently performed and compiled by Scriba et al. [45]. 

Several reviews have assembled the advances on preparation and evaluation of this type of CSPs over the years [43,45,49,50,51,52,57,58,59,80,85,86,87,88,89,90,91,92,93]. Nevertheless, this research field is always evolving being the most recent polysaccharide-based CSPs presented on Table S1 (supplementary material). 

Recent developments on polysaccharide-based CSPs comprise different approaches, with the general objective being the improvement of the enantioseparation performance. The strategies include the introduction of new polysaccharide derivatives (mainly new chitin and chitosan derivatives but also cellulose derivatives), hybrid selectors, and different chromatographic supports (monoliths, core-shell, microspheres), as well as the application of different methodologies for coating or immobilization procedures. Generally, for the new derivatives, the effect of different substituents on chiral recognition has been discussed [94,95,96,97]. Additionally, the effect of mobile phase composition on enantioseparation was also explored [94,95,96,98,99].

**Figure 2:**
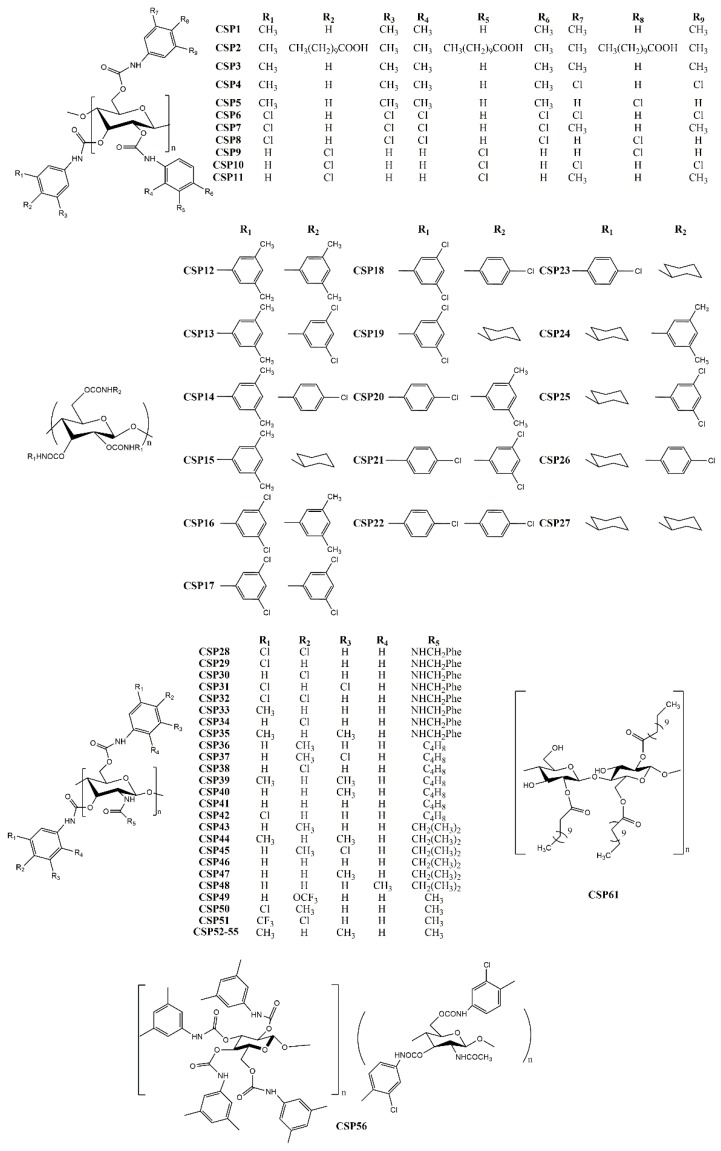
Chemical structures of polysaccharide-based CSP1–56 and CSP61.

Recently, Han et al. [94] developed two CSPs, using a derivative of cellulose *tris* (3,5-dimethylphenylcarbamate) (CSP1) and the same derivative functionalized with carboxylic acid (CSP2) (Figure 2). They concluded that a large variety of substituents could avoid the chiral recognition properties of the cellulose derivatives, reducing the performance of the CSP. The best chromatographic results were obtained for *trans-*stilbene oxide, with α and Rs values of 1.84 and 9.59, respectively. 

Shen et al. [100] synthetized cellulose derivatives with different combination of carbamate substituents and prepared 25 new CSPs (CSP3–27) (Figure 2). The effect of the carbamate substituents at 2,3-positions and 6-position of the glucose moiety were the main focus of the study. It was found that the chiral recognition properties of the CSPs comprising derivatives with two different phenylcarbamates were higher than if CSPs only had one substituent. The resolution was improved by the presence of different carbamate substituents, suggesting that the chiral recognition was dependent on the electronic properties, position and number of substituents of the glucose unit [100]. The highest separation factor obtained by using these recent CSPs was 2.87, for Pirkle alcohol.

Chitin and chitosan-based CSPs have received particular attention in the last few years [51]. Through continuous efforts to develop effective CSPs, other recent reports describing the use of chitin [101,102] and chitosan [95,96,97] derivatives are rising, with the carbamates as one of the most studied [49]. The growing interest in these polysaccharides comes from the fact that they have low solubility, which allows the use of a wide variety of mobile phases [52]. The influence of substituents on chitin and chitosan derivatives was also investigated. For some analytes, these CSPs possessed an enhanced chiral recognition when compared to cellulose and amylose derivatives, which may be attributed to the variety of solvents that can be used [103].

Tang et al. [97] developed eight CSPs (CSP28–35) comprising chitosan 3,6-*bis*(arylcarbamate)-2-(*p*-methylbenzylurea) with diverse substituents in the aromatic rings of the carbamates as well as in the amide group (Figure 2). Selectors with electron-donating substituents demonstrated a higher ability of enantioseparation. Previous reports emphasized that an electron-donating substituent at the 4-position of the aromatic ring was beneficial for the chiral separation [44]. Despite the selectors with 4-methyl substituent and 3-chloro-4-methyl portion presented a superior enantioseparation, the highest resolution (Rs = 18.1) and separation factor (α = 6.72) were obtained by the CSP with 3,5-dimethyl substituent [97]. 

In another study, Zhang et al. [95] prepared seven CSPs (CSP36–42) comprising derivatives of chitosan *bis*(phenylcarbamate)-(*N*-cyclobutylformamide) (Figure 2). The same substituent on different positions resulted on modifications on the suprastructure of the selector leading to different size of cavities, for example, due to different electronic effects. The obtained CSPs proved to have considerable stability on different solvents and a good enantiorecognition, allowing the researchers to obtain a separation factor of 8.64 for voriconazole [95].

Other new chitosan-based CSPs were developed, in this case, comprised of derivatives of chitosan (*bis*(methylphenylcarbamate)-(isobutyrylamide)) (CSP43–48) (Figure 2) [96]. The introduction of some substituents on specific positions of the aromatic ring linked to the carbamate were favorable for enantioseparation, such as methyl substituents. Additionally, the low solubility of chitosan was proved to be an advantage for the solvent tolerance and good enantioresolution performance achieved. As an example of its performance, high enantioselectivity and resolution were obtained for voricomazole, with α and Rs values 4.32 and 11.9, respectively [96]. 

Zhang et al. [102] synthetized derivatives of chitin using three different phenyl isocyanates (4-trifluoromethoxy, 3-chloro-4-methyl, 4-chloro-3-trifluoromethylphenylcarbamate) to develop three CSPs (CSP49–51) (Figure 2). All CSPs were applied for enantioseparation of tadalafil and its intermediate, demonstrating great enantiorecognition potential, with resolution and separation factor values of 4.72 and of 2.15, respectively [102]. 

Mei et al. [101] derivatized natural and regenerated chitins with 3,5-dimethyphenyl isocyanate, to prepare CSP52 and CSP53–55, respectively (Figure 2), with the difference between them only related to the raw material. The regenerated chitins were obtained from natural chitins, after a treatment with acetic anhydride, showing a more promising performance. They pointed out that CSP prepared from selectors with lower molecular weight provided an improved resolution [101]. The best chromatographic results were obtained for voricomazole, with Rs = 11.7 and α = 3.06.

Another strategy was the development of hybrid selectors [98]. Hybrid selectors or biselectors comprise two different polysaccharide derivatives coated on a chromatographic support [104]. Zhang et al. prepared the CSP56, which comprised a biselector based on derivatives of amylose and chitin (Figure 2), combining the low solubility of chitin derivatives with the excellent chiral recognition properties of amylose derivatives [98]. The obtained CSP presented an improved resistance against organic solvents with high enantioselectivity, with a Rs value of 8.49 for mephobarbital, and an α value of 4.32 for 2-(5-chloro-2-((4-methoxybenzyl)-amido)phenyl)-4-cyclopropyl-1,1,1-trifluorobut-3-yn-2-ol.

Regarding the use and preparation of chromatographic supports, a new technique of encapsulation in organic polymer monolith was reported, by Fouad et al. [103], as an alternative preparation of the chromatographic support. They functionalized an organic polymer monolith with carbamylated amylose as selector to obtain the CSP57. The synthesis of the amylose derivative was described previously [105]. The encapsulation of amylose was described as an economic methodology and it allowed the conjugation of amylose with reversed-phase elution mode for several analytes [103]. This promising technique allowed good results with a maximum resolution of 2.80 and a separation factor of 3.90 for the testes analytes.

Another different approach for preparation of chromatographic support was reported by Li et al. [99], who functionalized core-shell silver particles with cellulose derivatives through coating and intermolecular polycondensation and developed CSP58. A synergetic effect between silver and cellulose was observed considering the high values of resolution (Rs = 2.61) and enantioseparation (α = 8.42). This CSP demonstrated a particular selectivity toward analytes having the functional group ketone [99].

In another study, Bezhitashvili et al. [106] reported the covalent immobilization of a cellulose derivative (cellulose-(3,5-dichlorophenylcarbamate)) onto the surface of core-shell particles to obtain CSP59. The synthesis of the cellulose derivative was described previously [77]. The click chemistry was the synthetic methodology applied for the immobilization of the cellulose derivative to the chromatographic support [107]. The authors emphasized the short time of analysis achieved with baseline separations [106]. The highest separation factor obtained was 15.3 with a resolution of 11.0 for 2-(4-methylbenzylsulfinyl)-benzamide. 

Huang et al. [108] developed a new methodology for coating cellulose *tris*(3,5-dimethylphenyl carbamate) derivative on silica microspheres, without any surface pre-treatment since no aggregation occurred, and prepared CSP60 (range of pore size 10–150 nm). The synthesis of the cellulose derivative was previously described [109]. The silica microspheres with reduced size have functionalized polymeric beads being highly crosslinked [108]. This technique allowed a high-loading of the chiral selector and the obtained CSP provided a good performance, being the best separation factor of 2.41 for 2,2,2-trifluoro-1-(9-anthryl) ethanol. 

The CSP61 (Figure 2) was reported by Vieira et al. [110] using a technique of thermal immobilization of cellulose dodecanoate on silica particles without the use of chemical reagents. Despite of the absence of a chemical reagent during the procedure of immobilization, the selector was strongly linked to the chromatographic support allowing an exceptional selectivity. Some advantages of the immobilization technique were highlighted, including its low cost and eco-friendly feature [110]. The separation factor obtained was 3.10.

Besides the recent developments on polysaccharide-based CSPs, it is important to emphasize that this type of CSPs also cover a wide range of recent applications [111,112,113]. For example, Padró et al. [89] recently reviewed applications of polysaccharide-based CSPs in different fields. Studies comparing the enantioresolution performance of coated and covalently immobilized CSPs based on polysaccharides were also found [114]. 

Additionally, the influence of mobile phase is a common focus in several studies [81,115].

### 2.2. Protein-Based CSPs

The intrinsic ability of chiral recognition by enzymes, plasma proteins and receptors inspired the application of proteins in enantioseparation techniques [43]. Proteins are complex structures with a large surface area comprising a variety of stereogenic centers and different binding sites, which allow multiple possibilities of intermolecular interactions with small molecules [55]. The first application of a protein as CSP was reported in 1973, describing the separation of tryptophan enantiomers using a bovin serum albumin (BSA)-sepharose CSP [116]. After this first report, many CSPs based on proteins have been developed, with the most used proteins the human serum albumin (HSA), α_1_-acid glycoprotein (AGP), crude ovomucoid (OVM), and cellobiohydrolase I (CBH I) [86]. All these proteins as chiral selectors have been well documented for chromatographic enantioseparation for a wide range of chiral compounds and for binding affinity studies [117]. 

Proteins as CSPs are applied on affinity and pharmacokinetic studies since they can mimic the in vivo systems [118], being this feature very important in drug discovery [43]. The possibility of using aqueous or aqueous-organic as mobile phases was pointed out as other advantage of protein-based CSPs considering its compatibility with mass spectrometric detection. The disadvantages of this type of CSPs are the low capacity and efficiency. Moreover, the possibility of denaturation of protein limit the ranges of pH, ionic strength, temperature, and organic modifier composition of mobile phase [88], which is a result of its reduced chemical and biochemical stabilities. 

HSA is the most applied protein-based CSP, and it is used predominantly on studies of drug-protein binding [43]. In separation techniques, it is applied for weakly acidic and neutral compounds [119] as well as zwitterionic molecules [120]. For preparation of this type of CSPs, the protein can be physically adsorbed onto the packing material or it can be covalently bound [121]. 

A number of reviews on CSPs based on proteins have appeared over the years, focusing on their developments and applications [43,45,50,85,87,88,118,120,121,122]. Recently, Bocian et al. [117] briefly reported several studies related to protein-based CSPs, including the most common (HSA and AGP) as well as the more unusual, namely, avidin and fatty acid binding proteins. The developed strategies presented on that review were mainly related with the chromatographic support as the introduction of monoliths. The introduction of new selectors was also described [117]. Bertucci and Tedesco [42] recently reviewed the advances concerning the HSA as chiral selector highlighting the application of competitors for a particular binding site of HSA as the greatest advance. Scriba et al. [45] compiled some studies, focusing on the understanding of the binding sites and the main interactions between protein-based CSPs with diverse analytes, by molecular modeling.

The most recent protein-based CSPs was not reported in those reviews; their chromatographic performance are presented on Table S2 (supplementary material). It was found that different proteins as new selectors were not introduced. Nevertheless, diverse techniques of preparation of chromatographic support, namely new techniques of immobilization [42,117,123], entrapment [124], or the application of monoliths [125] have been reported attempting to overcome the stability problems of the proteins.

Proteins are usually immobilized on silica and its pore size can be defined in order to optimize the separation. Matsunaga and Haginaka [126] immobilized AGP on silica particles with different sizes, 5, 3 and 2.1 µm (CSP62–64). The use of this protein as chiral selector was reported, in 1985, by Hellerstein et al. [127]. In another study, Bi et al. [124] entrapped the same protein on a silica support with 100 Å and 300 Å (CSP65–66). Relatively to the first study mentioned, the resolution and efficiency of CSPs with lower particle size were superior. As an example, an excellent resolution was obtained for benzoin (Rs = 14.2) [126]. In the latter study, the described methodology using CSP65–66 proved to be an alternative to high-throughput screening and analysis of biological interactions due to the good affinity results, a maximum of 2.10 × 10^6^ M^−1^ [124].

Matsunaga and Haginaka [128] also studied the effect of particle size, on the efficiency of the column, with cellulase as chiral selector (CSP67). The first application of cellulase as chiral selector was reported by Vandenbosch et al. in 1992 [129]. As in the previous studies, the column with the lowest particle size provided the greatest efficiency and enantioselectivity [128], with a resolution value of 10.7 for propranolol, for example.

Zheng et al. combined a covalent immobilization process with a cross-linking/modification methodology, using HSA as chiral selector, to achieve CSP68. The aim of this new immobilization strategy was to enhance the protein retention [130]. The CSP obtained presented a high binding affinity for warfarin, with a maximum affinity constant of 2.60 × 10^5^ M^−1^. 

A polyclonal antibody CSP (CSP69) was developed by Bi et al. [131] through an alternative methodology, which consisted of the isolation and immobilization of the selector presented on a serum sample (on-line immunoextraction). This methodology was previously described by Matsuda et al. [132]. Once again, it was found that it could be an alternative to the traditional immobilization methodologies since it is not necessary extra steps of protein purification and immobilization [131]. The stability and the robustness of the CSP were also highlighted [131]. This methodology allowed the preparation of a CSP with considerable affinity. For example, a binding affinity value of 90.0 × 10^6^ M^−1^ was obtained for disopyramide.

A new protein-based CSP was reported by Fedorova et al. [133] using a different adsorption methodology, which consisted of BSA adsorbed on eremomycin and grafted on silica (CSP70). An improved resolution, with a good resolution (Rs = 2.14) was provided, in comparison with a CSP comprising only eremomycin. 

One of the most recent developments concerning this type of CSPs was the functionalization of monoliths with proteins. Monoliths are based on silica and present the advantage of optimizing the proportion of monomers and cross-linkers. This optimization enables the control of the average size of the throughput channels and the porous [134]. Monoliths can be prepared with different materials and techniques; the advantages comprise a superior flow as well as an enhanced mass transfer resulting on a more efficient separation [121]. Pfaunmiller et al. [125] immobilized HSA on monoliths to obtain CSP71–72. The main objective was to optimize the amount of protein that could be immobilized. As a consequence, the prepared monoliths allowed an improvement on all chromatographic parameters [125]. 

The recent applications described for this type of CSPs are more diversified than the developments and some of them are related with the optimization of the chromatographic conditions [133,134,135]. Nevertheless, the number of publications describing the use of protein-based CSPs has been decreasing over the years [117,136]. Binding affinity studies between drugs and proteins and drug-protein interactions were also found [137,138,139].

### 2.3. Cyclodextrin-Based CSPs

The first application of cyclodextrins as CSPs was described by Armstrong and DeMond in 1984 [140]. Since then, several cyclodextrin-based CSPs have been reported [137,140,141,142]. Cyclodextrins consist on cyclic oligosaccharides [88]; this type of macrocycles can be divided into three classes, α, β, and γ [43]. The structure of a cyclodextrin consists on a truncated cone [43] with an interior non-polar cavity and free hydroxyl groups located on larger and tiny edges [143]. The hydroxyl groups can be derivatized with diverse polar or apolar substituents [55], which can influence the conformational flexibility of a given cyclodextrin, modifying the size of its cavity and creating additional binding sites [43]. 

The chiral recognition mechanism is typically based on the formation of an inclusion complex between the analytes and the internal cavity of the cyclodextrin [43]. Additionally, the analytes can establish different types of interactions with the exterior side, including dipole-dipole, hydrogen-bond, ionic, π-π, or London interactions [120]. Cyclodextrins present a considerable number of stereogenic centres, which also contributes to enantiorecognition [55].

Cyclodextrin derivatives can be prepared through physical coating or covalently bonding to a chromatographic support [144]. Covalent bonding of cyclodextrin derivatives is the most applied methodology, since it provides a powerful and resistant linkage to the chromatographic support. The most common linkers are ether, amino, and urea. Recently, a triazole linker was also described [144].

The high stability of this type of CSPs allows the use of an extensive variety of solvents as components of mobile phases, with a wide range of polarities, affording an efficient enantioseparation for different analytes [55]. This type of CSPs can be applied in multimodal elution conditions [142]. 

Several reviews have been devoted to the developments and applications of cyclodextrin-based CSPs [43,45,85,87,88,140,142,144]. Additionally, Guo et al. [145] reviewed the most recent developments concerning on cyclodextrin functionalized monolithic columns. 

Studies related to chiral recognition mechanisms of this type of CSPs using diverse methodologies, such as nuclear magnetic resonance, docking, or molecular modeling, were also addressed to understand the molecular interactions as well as the effect of some chromatographic conditions, such as pH, temperature, or organic modifier, in the enantioseparation [146,147].

The recent cyclodextrin-based CSPs and the evaluation of their chromatographic performance are described on Table S3 (supplementary material). The most recent developments are comprised mainly of the introduction of new derivatives and application of different methodologies of immobilization to the chromatographic support. The preparation of hybrid CSPs to enhance the interactions between the analyte and the stationary phase was also emphasized. It was found that the majority of the new cyclodextrin-based CSPs were prepared based on the most widely used cyclodextrin as CSP, i.e., the β-cyclodextrin [148,149,150,151]. Moreover, the immobilization strategy of the chiral selector on the chromatographic support was, mainly, by click chemistry [149,150,151,152]. The main advantages of this approach are the mild reaction conditions and the enhanced tolerance of the CSPs to solvents and the range of pH values [153]. The introduction of new methodologies to prepare the chromatographic support was also focused, including the preparation of hybrid supports [154,155], the introduction of monoliths [156] and new chromatographic supports [157], or using a different technique to prepare the support [158]. 

**Figure 3:**
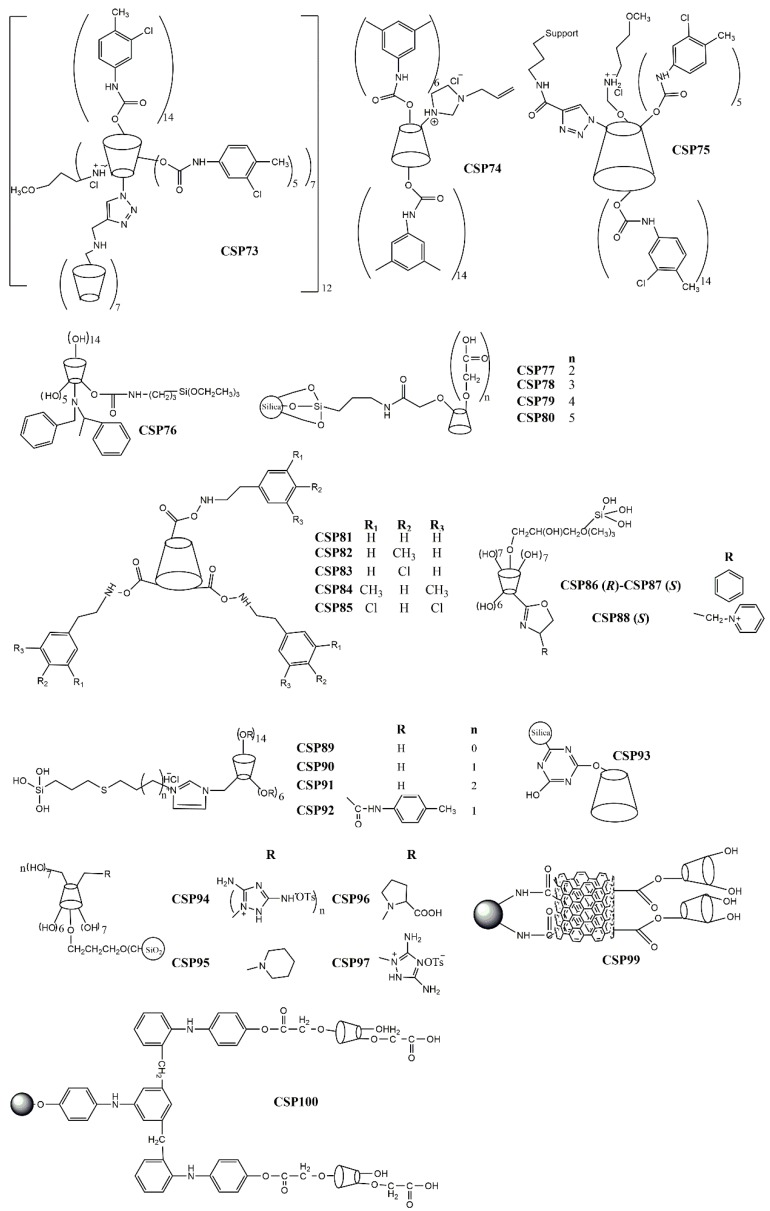
Chemical structure of cyclodextrin-based CSP73–100.

Zhou et al. [152] reported the linkage of a C6-disubstituted cationic β-cyclodextrin onto an alkynylated β-cyclodextrin bonded to a silica support to afford the CSP73 (Figure 3). The obtained bilayer cationic β-cyclodextrin presented a remarkable enantioselectivity for the tested analytes. As an example of its excellent enantioseparation and resolution, α and Rs values of 2.39 and 4.40, respectively, were obtained for 4-(chlorophenyl) propyl ester [152].

Tang et al. [151] resorted to thiol-ene click chemistry to prepare a sulfoether-bridged cationic per(3,5-dimethyl) phenylcarbamoylated-β-cyclodextrin-based CSP (CSP74) (Figure 3) being able to establish π-π interactions and hydrogen bonding interactions with the tested analytes. Its enantiorecognition ability was demonstrated by a separation factor of 1.70 and resolution of 6.03 for 3-(chlorophenyl) propyl ester.

Zhou et al. prepared a perphenylcarbamate β-cyclodextrin chloride linked by click chemistry to an alkynyl silica support to obtain the CSP75 (Figure 3) [159]. After evaluation of its enantioseparation performance using diverse analytes, they concluded that the introduction of the 3-methoxypropylammonium substituent promoted favorable intermolecular interactions with the analytes. In addition, it was suggested that the mobile phase could cause steric hindrance which prevented the establishment of interactions that were crucial for enantiorecognition [159]. The performance of the CSP was promising with a maximum resolution value of 9.84 and a separation factor of 2.76 for 7-methoxyflavanone and 6-methoxyflavanone, respectively.

A new *N-*benzyl-phenethylamino-β-cyclodextrin was synthetized and bonded to mesoporous nanoparticles of silica obtaining the CSP76 (Figure 3) [149]. The new CSP demonstrated to have a superior flexibility and stability, in comparison with the native β-cyclodextrin-based CSP, being obtained through a more economic process [149]. Relatively to its performance, the higher separation factor and resolution values were 1.30 and 1.97, respectively, for carvedilol.

Four new cyclodextrin-based CSPs (CSP77–80) (Figure 3) were developed by chemical bonding of carboxymethyl-β-cyclodextrin derivatives to silica gel by an amidation reaction on aqueous solution [160]. The carboxymethyl moiety provided additional interactions with the tested analytes, in comparison with the native β-cyclodextrin demonstrating a superior enantioselectivity and resolution [160]. For example, an excellent separation factor value (α = 6.08) was achieved for methyl 2-amino-3-(3-(methylsulfonyl)phenyl)-propanoate hydrochloride. Moreover, for 1-((benzyloxy)carbonyl)-4-hydroxypyrrolidine-2-carboxylic acid, the resolution value was 9.56 [160].

The effect of different substituents on CSPs has also been investigated. Chen et al. [148] synthetized β-cyclodextrin derivatives with a phenylcarbamate moiety with different patterns of substituents, which were subsequently immobilized onto the silica gel through intermolecular polycondensation of the triethoxysilyl groups (CSP81–85) (Figure 3). They reported that the presence of an aromatic ring with electron-withdrawing groups on the β-cyclodextrin improved the chiral recognition for analytes with electron-donor groups since the number of possible π-π interactions was superior [148]. The hydrogen-bond interactions between the carbonyl group or nitrogen of the analytes and the amino group of phenylcarbamate of cyclodextrin-based CSP were also improved [148]. Relatively to the performance of the CSPs, the highest separation factor value achieved was 2.87 for Pirkle alcohol.

Li et al. [150] arrived at similar conclusions after preparing oxazolinyl-functionalized β-cyclodextrins covalently bonded to silica support (CSP86–88) (Figure 3). They described that analytes with electron-donating or hydrogen-bonding groups were easily enantioseparated due to a higher number of π-π and hydrogen-bonding interactions [150]. CSP88 was more suitable for enantioseparation of polar compounds since it promoted electrostatic interactions due to the presence of an ionic group [150]. Additional factors that could influence the enantioseparation performance of the CSPs, such as the spacer length, selector concentration, and rim functionalities, were also investigated [150]. A reduced surface concentration and a superior flexibility of the spacer decreased the enantioselectivity, since it weakened the interactions between the selector and the analyte [150]. Additionally, a superior selector concentration could be beneficial for enantioseparation of some racemates. The performance of the CSP was promising with an excellent resolution for ketoprofen (Rs = 22.0) and a separation factor value of 15.5 for loxoprofen.

The same group developed four thioether bridged cationic cyclodextrin-based CSPs (CSP89–92) (Figure 3) and the influence of the spacer length, selector concentration, and rim functionalities on the performance of the CSP were studied [161]. In this case, it was found that CSPs comprising a spacer with a superior length could compromise their ability of enantiorecognition; however, a higher concentration of the selector was positive [161]. The higher resolution value achieved was 12.7 for 4-nitrophenyl propyl oxide, and the separation factor value was 3.30 for styrene oxide.

Regarding the development of hybrid CSPs, a spherical β-cyclodextrin-silica hybrid CSP (CSP93) (Figure 3) was reported by Wang et al. [154] highlighted by the presence of multiple functional groups, which expanded the spectrum of possible interactions with analytes. The β-cyclodextrin derivative was introduced into the pore channels and pore wall framework, and the linker was attached just into the pore channels [154]. Both the interior and exterior of the CSP participate in the process of the enantiorecognition. For example, separation factor and resolution values of 1.63 and 4.65 were obtained for diclofop and mandelonitrile, respectively.

Regarding the use of new materials as chromatographic supports, a modification of the most common chromatographic support was performed by Zhao et al. [155] to obtain new CSPs. The modified silica gel was named hydride silica, and its surface was covered by silica-hydrogen bond instead of silica-hydroxyl. The hydride silica presents a superior resistance to water, a reduced polarity, and an improved separation rate and stability and it can be used with a wide variety of solvents [155,162]. Zhao et al. [155] prepared polar group derivatives of β-cyclodextrin bonded to hydride silica to obtain CSP94–97 (Figure 3). The higher resolution value achieved was 9.31 for methyl (2*R*,3*R*,4*S*,5*R*)-5-(4-fluorophenyl)-4-nitro-3-phenyl-3-(trifluoromethyl)-pyrrolidine-2-carboxylate; the best separation factor value was 3.65 for methyl (2*R*,3*S*,4*S*,5*R*)-5-(4-fluorophenyl)-4-nitro-3-(*p*-tolyl)-pyrrolidine-2-carboxylate.

Ghanem et al. [156] used a different strategy to prepared new cyclodextrin-based CSPs. They encapsulated the trimethylated-β-cyclodextrin to a polymeric monolithic, to obtain a superior surface area, reduced pore size, and enhanced total pore volume, and developed the CSP98. They also studied the physical characteristics of the CSP to established relationships with the potential of enantiorecognition and concluded that a superior concentration of the selector improved the enantioseparation [156]. The CSP98 demonstrated a suitable mechanical and thermal stability as well as reproducibility [156] with a maximum resolution value of 2.51 for flavanone, and a separation factor of 1.42 for carprofen.

A different chromatographic support was also proposed by Qiang et al. [157], who described a β-cyclodextrin CSP based on graphene oxide (CSP99) (Figure 3), which was covalently linked to amino silica gel by an amide bond. The graphene oxide and cyclodextrin presented a synergetic effect for enantiorecognition being the hydrogen bonding and π-π interactions the main interactions between the CSP and analyte. The CSP99 was also applied for hydrophilic interaction chromatography. Regarding the chromatographic results, a separation factor of 38.8 was achieved for equol, and a resolution value of 2.17 for 1-phenylethanol [157].

A light-assisted preparation of carboxyl methyl β-cyclodextrin-based CSP (CSP100) (Figure 3) was described by Tang et al. [158] who used ultra-violet light to link the chiral selector to silica, which promoted the modification of ionic bonds into covalent bonds. This technique proved to be eco-friendly and efficient [158]. The morphology and chemical composition of CSP100 was characterized. Moreover, it was concluded that its enantiorecognition ability was dependent of hydrogen bonding and dipole-dipole interactions [158]. The maximum resolution value achieved was 8.04 for chlortrimetron.

### 2.4. Macrocyclic Antibiotic-Based CSPs 

Macrocyclic antibiotics are the second most versatile group of CSPs, after polysaccharides; their planning was inspired by cyclodextrins. The first report of macrocyclic antibiotics as CSP was in 1994, describing the application of vancomycin, thiostrepton, and rifamycin B as CSPs [163].

Macrocyclic antibiotics are divided into four groups: ansamycins, polypeptides, glycopeptides, and aminoglycosides [164]. Ansamycins comprise an aromatic unit linked to an aliphatic chain, and their classification is based on the aromatic moiety. If the aromatic unit is a naphthalene or naphthoquinone, it is denominated naphthalenic ansamycin, while if it is a benzene or benzoquinone, it is a benzenic ansamycin [164]. The most common ansamycins used as CSPs are rifamycins B and SV; the first one is enantioselective for cationic compounds and the second for neutral and anionic [165]. Polypeptides have few aromatic ring units while aminoglycosides do not have this type of structural feature [165]. Only one polypeptide is used as CSP, thiostrepton, whereas aminoglycoside class comprises more CSPs, such as fradiomycin, kanamycin, and streptomycin [166].

Glycopeptides are the most promising class of macrocyclic antibiotic-based CSPs, including avoparcin, ristocetin A, teicoplanin, vancomycin, and derivatized analogues from vancomycin, among others [165]. The chemical structure of glycopeptides consists on a glycosylated cyclic or polycyclic peptide. The central framework is a heptapeptide, in which five of the seven amino acid residues are common to all glycopeptides [164]. Glycopeptides have some flexibility due to the possibility of rotation of sugar groups [165].

The structure of macrocyclic antibiotics allows a variety of interactions with the analytes, such as hydrophobic, π-π, dipole-dipole, hydrogen-bond, electrostatic, ionic, and Van der Waals interactions [166], being either attractive or repulsive. It is possible for the formation of inclusion complexes to occur [167]. The high number of stereogenic centers in their structures also contributes for its high capacity of chiral recognition [55]. Nevertheless, the chiral recognition mechanism of this type of CSPs it is not currently quite understood [43]. 

The chromatographic support of this type of CSPs is, predominantly, silica gel [164]. Macrocyclic antibiotic-based CSPs are able to operate in all chromatographic elution modes [88]. Besides that, the macrocyclic antibiotic-based CSPs provide a complementary enantioselective profile [167].

Over the years, the developments carried out to obtain diverse macrocyclic antibiotic-based CSPs as well as their applications have been compiled [43,45,85,87,88,164,165,167,168]. Additionally, some authors focused their studies on the mechanism of chiral recognition [169,170]. 

The most recent reports related to new macrocyclic antibiotic-based CSPs as well as their chromatographic parameters are presented in Table S4 (supplementary material). The developments did not comprise the introduction of new antibiotics as chiral selectors but rather the use of new chromatographic supports, specifically the use of silica particles with sub-2-μm size. In fact, they are mainly based on the preparation of new teicoplanin and vancomycin-based CSPs by reducing the size of the packaging material [171,172,173]. The main objective was the improvement on chromatographic performance by reduction on analysis time and enhance of resolution and enantioselectivity.

Min et al. [171] described the preparation of a teicoplanin-based CSP bonded to sub-2 μm superficially porous particles (CSP101). The main focus was to avoid aggregation and to uniformize the size distribution, enhancing the surface area. The high resolution and enantioselectivity obtained in a short time of analysis were highlighted. The maximum resolution value was 5.60 for methionine, and the separation factor was 9.40 for norvaline.

Ismail et al. [172] developed a teicoplanin-based CSP with a sub-2 μm chromatographic support; however, in this case, they used totally porous silica particles (CSP102). They pointed out the flexibility of the CSP to operate on different elution modes. The selectivity, efficiency, and the short analysis time on ultra-high-performance liquid chromatographic (UHPLC) were also emphasized [172]. Its performance was promising achieving a resolution value of 10.7 for alanine, and a separation factor of 3.45 for mandelic acid.

Vancomycin was bonded to sub 2-μm diol hydride-based silica particles by Rocchi et al. [173]. Four new CSPs were developed (CSP103–106) with the same main objective: reduction of analysis time. It was inferred that this technique could be applied to other chiral selectors due to the promising results [173]. The maximum resolution value was 3.36 and the separation factor was 2.69 for haloxyfop. 

Despite the reduction of particle size of the support, new materials were introduced as chromatographic support. Recently, Xu et al. [174] described the preparation of a vancomycin-based CSP through the combination of monoliths and polymeric cross-linking (CSP107). The new CSP possessed a good mechanical stability, permeability, and enantioselectivity [174]. The influence of some chromatographic conditions was also investigated. The performance of the CSP was satisfactory, with a resolution value of 1.47 for salbutamol, and a separation factor of 1.23 for carteolol. Hellinghausen et al. [175] prepared the CSP108 through the prior synthesis of vancomycin, by Edman degradation, and further binding it to superficially porous particles through a primary amine group of vancomycin, which resulted from the removal of an *N*-terminus leucine residue. The CSP108 presented promising results with a good resolution and separation factor values for 2-amino-2-phenylbutyric acid (Rs = 2.70 and α = 1.57).

A vancomycin-based CSP was recently prepared (CSP109) by a photochemistry-based method [176]. Additionally, the influence of flow rate, elution mode, buffer, and the mass of analyte were also investigated. The addition of 2-propanol, buffer and an increase on analyte mass improved its enantioresolution performance, since π-π interactions were superior. The chromatographic performance was good, with a maximum resolution of 3.08 and a separation factor of 4.23.

It is also important to highlight that the complementary behavior of the different macrocyclic antibiotic-based CSPs continues to be a subject of great relevance. In fact, several recent studies can be found in literature [166,167,177]. Most of them compared the enantioresolution performance of teicoplanin and teicoplanin aglycone CSPs [166,167] or of vancomycin and teicoplanin CSPs [177].

### 2.5. Brush-Type or Pirkle-Type CSPs

Brush-type or Pirkle-type CSPs were introduced in 1979, when Pirkle and House described the development and application of a chiral fluoro alcoholic CSP able to enantioseparate diverse classes of analytes [178]. 

Neutral synthetic chiral low-molecular mass molecules are the base of this type of CSPs [43]. These molecules should promote donor-acceptor interactions as a hydrogen-bond, π-π, or dipole-dipole, in addition to attractive and/or repulsive Van der Waals interactions [45]. As they comprise small molecules as chiral selectors, the mechanism of chiral recognition is, frequently, based on the “three-points” model, which refers that the establishment of at least three interactions between one of the enantiomers to be resolved and the CSP are essential for chiral recognition [179]. 

The chiral selectors are usually covalently linked to a silica support, which can have monosubstituted or trisubstituted silane groups, through a spacer [55]. Over several years, Pirkle’s group has developed successive generations of CSPs [180], based on the principle of reciprocity [181] and on chromatographic [182,183] and spectroscopic [184,185] methods to understand the chiral recognition mechanisms. Among them, Whelk-O1 CSP, created by a rational approach, is the most applied and versatile CSP in both academic and industrial fields [186].

Initially, the preferred elution mode was the normal phase since it provides a favorable environment for the interactions needed to enantioseparate the analytes [88]. Nevertheless, this type of CSPs can also be used in polar organic and reversed-phase elution modes [187,188,189].

The advantages inherent to this type of CSPs are the compatibility with a wide range of solvents used as mobile phase, the stability to temperature and pressure, the considerable loading capacity and the possibility to be easily scaled up to preparative chromatography [190]. Another key advantage is the possibility of switching the configuration of the chiral selector and to use the inverted configuration column approach [43]. Its kinetic performance is reasonable and the fact that the structure of the chiral selector is relatively “simple” allows an easier knowledge of the chiral recognition mechanisms as well as a consecutive optimization of the selector [191]. Pirkle-type CSPs are characterized by their diversity and versatility since it is possible to use a variety of different small molecules as chiral selectors as well as introduce different substituents that can improve enantioselectivity. In addition, they can be highly specific for certain types of chiral compounds [120].

Pirkle-type CSPs have evolved over the years. Certain types of CSPs have more reported progresses, mainly due to the possibility of use of a wide variety of small molecules as chiral selectors. Several Pirkle-type CSPs can be found in literature comprising chiral selectors closely related to the original Pirkle’s group CSPs and others structurally different [43,45,85,87,88,180,186,190,191]. Recently, a literature survey made by our group covering the report on Pirkle-type CSPs developed during the last 17 years was published [190]. We described 226 new CSPs, including a wide diversity of small molecules as chiral selectors, including amine, amino alcohol and amino acids derivatives, peptides, drugs, selectors based on natural products, and xanthone derivatives, among others [190]. 

The recent developments of this type of CSPs also include the use of new chromatographic supports, such as monolith supports, core-shell particles, or particles with a reduced size (sub 2-µm). The reduction of particle size enables the adaptation of Pirkle-type CSPs to UHPLC, the first ones to be converted, which are associated with the inherent advantages such as the reduction of analysis time and quantity of the solvent, improved efficiency, and enantioresolution [192,193]. Regarding the use of core-shell particles, it was found that the packaging with this type of material or, alternatively, with fully porous particles led to differences on chromatographic performance. The diffusion on core-shell particles is lower than in fully porous particles, which is especially beneficial for large analytes, since it prevents a decrease on efficiency due to an inefficient mass transfer. The distribution of particle size of core-shell particles is nearly unimodal, which increases efficiency on enantioseparation of small analytes [194,195].

Different synthetic methodologies to obtain the chiral selectors as well as for its immobilization on the chromatographic support were also introduced. The synthesis of biselectors was another approach [190]. The most recent Pirkle-type CSPs and the chromatographic parameters obtained after evaluation of their enantioresolution performance are presented on Table S5 (supplementary material). 

**Figure 4:**
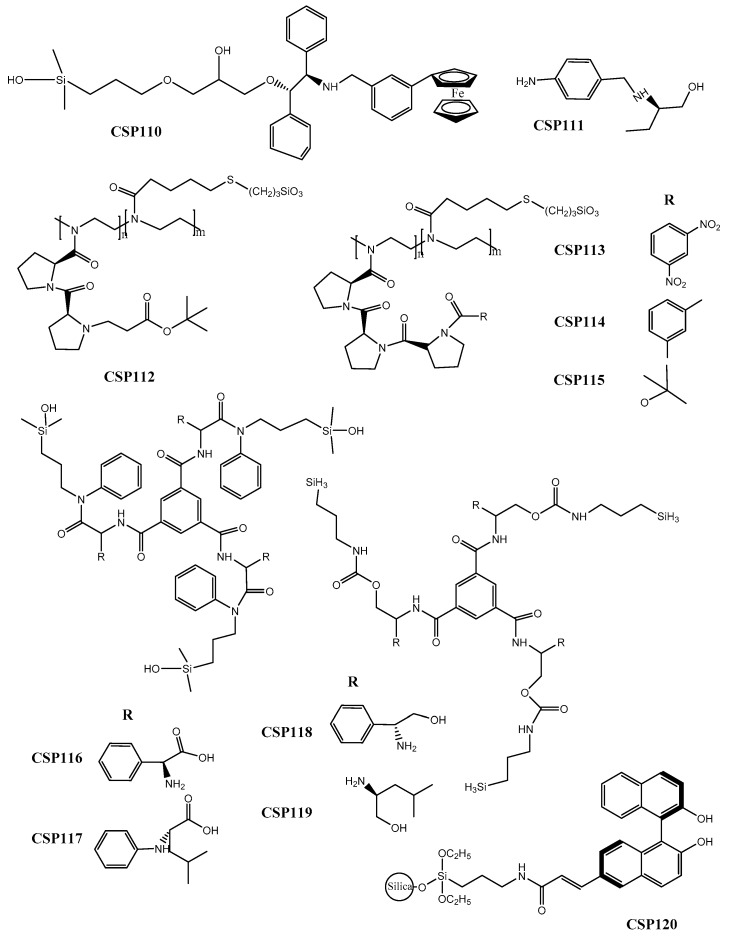
Chemical structures of Pirkle-type CSP110–120.

Qiao et al. [196] developed a CSP based on *N*-ferrocenyl benzoyl-(1*S*,2*R*)-1,2-diphenyl ethanol as a chiral selector (CSP110) (Figure 4). The conjugation of a cyclopentadienyl carbon ring with an aromatic ring demonstrated to improve enantioselectivity. The chiral recognition mechanisms were also explored, revealing that hydrophobic, hydrogen-bond, π-π, and dipole-dipole interactions between the chiral ferrocene CSP and acidic and basic groups of the analytes were crucial. The performance of the CSP was promising achieving a maximum resolution value of 4.13 and a separation factor of 2.43 for 3-nitrophenol.

Çakmak et al. [197] synthetized an aromatic amine derivative of (*R*)-2-amino-1-butanol for the application as chiral selector of a new CSP (CSP111) (Figure 4). In the same study, they used docking, molecular dynamics simulation, and quantum mechanical computation methods to characterize the mechanisms of chiral recognition. The performance of the new CSP was good with a high resolution value of 3.85 for 2-phenylpropionic acid, and a separation factor of 2.75 for mandelic acid.

Four new pseudopeptide-based CSPs were developed (CSP112–115) (Figure 4) inspired by the possibility of enantiorecognition ability of an organocatalyst [198]. It was found that the enantioselectivity of the CSPs was dependent of the degree of derivatization of diproline portion and of the length of polymeric chain. The chromatographic results were promising, achieving, for example, separation factor and resolution values of 9.80 and 2.89, respectively, for 1-phenylethan-1-amine and *N*-(1-(naphthalen-2-yl) ethyl)-3,5-dinitrobenzamide.

Additionally, in another recent work, derivatives of amino acids and amino alcohols as CSPs were prepared by Yu et al. [199], based on C3-symmetric CSPs (CSP116–119) (Figure 4). It was found that a phenyl group linked to amide was crucial for chiral recognition and, despite the chiral selectors did not possess a π-acidic or π-basic group, their performance was promising. For example, a separation factor value of 2.58 was achieved for 2-phenyl-2-pentanol. 

Wang et al. [200] synthetized the (*R*)-6-acrylic-binaphtol as chiral selector through addition of the acrylic group to the (*R*)-binaphtol and developed the CSP120 (Figure 4). The mechanisms of chiral recognition, the effect of the temperature and mobile phase composition were also discussed. It was found that the flexibility of the CSP and the π-π stacking event allowed the retention of the analytes without compromise the enantioseparation [200]. Regarding the chromatographic results, a separation factor value of 1.12 was achieved for 3,5-dinitro-*N*-(1-phenylethyl) benzamide. 

Along with the continuous developments of this type of CSPs, it is important to emphasize that a broad range of recent applications have also been reported [201,202,203,204,205].

### 2.6. Ion-Exchange-Type CSPs

Ion-exchange-type CSPs were introduced by Salvadori et al. in 1985, who described the application of cinchona alkaloids as CSPs [206]. Nevertheless, Lindner group developed the majority of this type of CSPs [207]. Ion-exchanger selectors can be subdivided into three groups: anionic, cationic, or zwitterionic [208]. 

The most common anion-exchangers as chiral selectors are cinchona alkaloids [45] and terguride [43]. Anion-exchanger selectors are appropriate for enantioseparation of acidic compounds; their enantioselectivity are attributed to the five stereogenic centers of the basic nucleus common to quinine and quinidine [209]. Cation-exchanger selectors are useful for enantioseparation of basic analytes, which are structurally based on chiral sulfonic or carboxylic acid compounds as selectors [45]. Zwitterionic selectors were introduced, more recently, by Lindner et al. [210] by merging key cation- and anion-exchange moieties in one single chiral selector [45]. Those CSPs can been applied for the enantioresolution of acid, basic, and amphoteric compounds [209]. Zwitterionic CSPs have overcome the main disadvantage of anion and cation-exchanger CSPs, since these two groups only separate enantiomers with opposite charge [210].

The chiral mechanism of recognition is mainly based on ionic interactions between the charged analytes and the opposite charged groups of the CSPs [208]. Hydrogen bonds as well as π-π interactions are also important for complex formation [45]. The ion-pairing of solvent controls the adsorption and retention of the analytes [43]. Polar-organic and reversed-phase elution modes are the preferential elution modes for this type of CSPs [43]. The retention and enantioselectivity are affected by the pH and the nature and concentration of acid or base added to the mobile phase [43].

The progresses resorting to this type of CSPs have been reviewed over the years [43,45,85,88,207,209,211,212,213]. Recently, Ilisz et al. [213] compiled the most recent developments concerning to anionic and zwitterionic-exchange-based CSPs, which are related to the application of different techniques of preparation of chromatographic support to attempt the optimization of chromatographic parameters. The most recent ion-exchanger-type CSPs and their chromatographic parameters are presented on Table S6 (supplementary material). The majority of the recent developments focused on quinine and quinidine derivatives as chiral selectors [214,215,216,217], the key structural moiety representative of anion-exchanger selectors.

**Figure 5:**
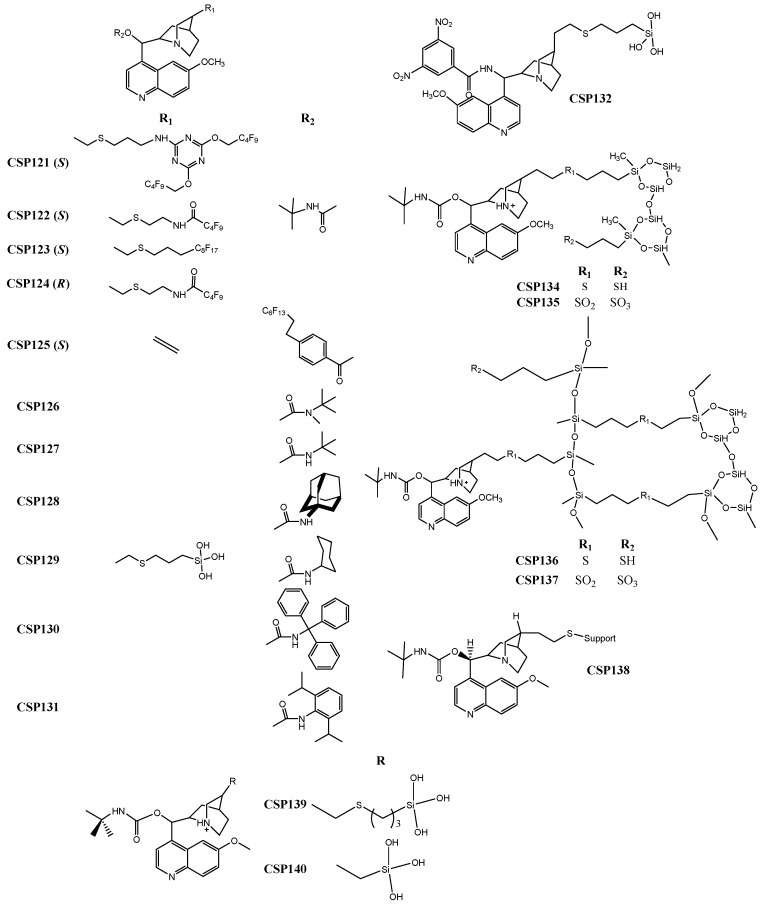
Chemical structures of Ion-exchange-type CSP132–140.

Todoroki et al. [214] developed a new technique to prepare new ion-exchange CSPs, specifically cinchona alkaloid-based quinine and quinidine-type fluorous-tagged-CSPs (CSP121–125) (Figure 5). The main objective was to improve the enantioseparation properties enabling a sensitive, selective, robust, and reproducible analysis methodology. The versatility of the new CSPs was another advantage, as it was capable to enantioseparate bulky, aromatic compounds, in addition to amino acids, such as threonine with a resolution value of 11.8, and asparaginine, with a separation factor of 4.56. 

The complementarity profile between anion-exchange-type CSPs was the focus of a recent study reported by Lämmerhofer et al. [218], who prepared several cinchona carbamate selectors with distinct carbamate residues to obtain CSP126–131 (Figure 5). Different structural moieties were introduced to enhance the possibility of complementary; for example, the introduction of bulky groups to create steric hindrance or aromatic rings to provide π-π interactions. The complementary accomplished with the new CSPs allowed the expansion of the enantioselectivity range. The performance of the CSPs was promising, achieving, for example, a separation factor of 17.0 for leucine.

De Martino et al. [216] reported the synthesis of an anion exchange hybrid selector, the 3,5-dinitrobenzoyl-9-amino-9-deoxy-9-epiquinine, to develop CSP132 (Figure 5). The strategy applied was the association of typical moieties of Pirkle-type selectors with key moieties of anionic-exchange-based selectors, enlarging the possibility of multiple interactions with the analytes. The performance of this hybrid CSP was promising, with separation factor and resolution values of 2.06 and 11.0, respectively, for diazepam *N*-oxide.

A new immobilization technique based on click chemistry, was described by Lämmerhofer et al. [219], who prepared a cross-linked *tert*-butylcarbamoyl quinine-based CSP (CSP133). The technique allowed achieving a CSP with reduced resistance to mass transfer and retention times, as well as an improved stability. During the optimization of the procedure, some features were discussed, such as the amount of polysiloxane, chiral selector, radical initiator, and reaction solvent, as well as reaction time and size of the chromatographic support particles [219]. The performance of the CSP was promising; for example, with separation factor and resolution values of 1.54 and 5.20, respectively, for *N*-[(9*H*-fluoren-9-ylmethoxy) carbonyl]-phenylalanine.

The same group also resorted to click chemistry to prepare other CSPs based on *tert*-butylcarbamoyl quinine (CSP134–137) (Figure 5) [215]. The optimization of the selector’s structure allowed the avoidance of non-specific interactions that could reduce chiral recognition [215]. The introduction of a sulfonic group afforded a reduction on the retention times and an improvement, in some cases, of separation factors since its negative charge provided electrostatic interactions, promoting an effect similar to the counterion effect [215]. The performance of the CSP was satisfactory achieving a maximum resolution value of 6.20 for *N*-(9-fluorenylmethoxycarbonyl)-phenylalanine and a separation factor of 1.66 for *N*-acetyl-phenylalanine.

The application of core-shell particles was another strategy. A new CSP based on *tert*-butylcarbamoylquinine selector (CSP138) (Figure 5) was described by the same group, to promote the enantioseparation of several proteinogenic amino acids [217]. Core-shell particles were introduced in order to improve the analysis time, which was a promising methodology for the bioanalytical area, since it could be combined with sensitive fluorescence detection or highly sensitive and selective mass spectrometric detection. The column presented a reasonable performance with good enantioselectivity and resolution. For example, α and Rs values of 1.55 and 4.08, respectively, were achieved for threonine [217].

Armstrong et al. [220] also resorted to core-shell particles to develop two new quinine-based CSPs (CSP139–140) (Figure 5) for ultrafast liquid and supercritical fluid chromatography. The new CSPs allowed fast analysis with high enantioselectivity and efficiency for the tested analytes. The performance of the new CSPs was promising, affording, for example, a maximum resolution of 25.5 and a separation factor of 14.5 for *N*-(3,5-dinitrobenzoyl)-leucine.

It is important to highlight that recent applications of this type of CSPs were diversified, with the focus, mainly, on the application of anion and zwitterionic-type CSPs with amino acids [221,222,223,224,225].

### 2.7. Crown-Ether-Based CSPs

Crown-ethers as CSPs were firstly described by Sousa et al. [226] for the enantioseparation of primary amine salts. Crown-ethers consist on macrocyclic polyethers, with a cavity of a specific size, being able to form complexes with analytes [227]. CSPs based on crown-ethers can be divided into two major groups: the crown- ethers comprising a 1,1′-binaphthyl group and those containing two tartaric acid groups [228]. The enantiomers of α-amino acids and primary amines may be separated by the first type of crown-ethers CSPs [229]; the second group can be applied for enantioseparation of primary and secondary amino compounds and non-amino compounds [229].

The mechanisms of chiral recognition are typically driven by triple hydrogen bonds established by an ionized ammonium group of the analytes and three oxygen of the CSP, leading to the formation of an inclusion complex [43]. The electron-donor oxygen particles are distributed on inside of the cavity of the crown-ether [88]. Steric hindrance from the substituents of the analyte ions and the functional groups of the crown-ethers can influence the enantioseparation [43]. Additional interactions are essential to complement the formation of the complex, including π-π, hydrogen-bond, and dipole-dipole interactions [230]. Mobile phases should be strong acidic aqueous solutions to achieve the total protonation of the amino group of the analytes [43]. Crown-ether-based CSPs can be obtained by a coating process or by immobilization [231]. To avoid the leaching of the CSP from the column and to allow the analysis of hydrophobic compounds the use of covalently bonded CSPs it can be preferable than the coated [232].

The developments of crown-ether-based CSPs have been revised over the years [43,45,85,87,88,228,229]. Hyun et al. [228] reviewed the most recent developments, related to both classes of crown-ethers as CSPs, highlighting techniques for the preparation of the chromatographic support or the protection of unreacted residues. 

Crown-ether-based CSPs have some restrictions related to the target analytes; however, their preparation and the chromatographic conditions can be modifier to improve the chromatographic results. The majority of the recent developments encompassed the use of different strategies for immobilization of the chiral selectors to the chromatographic support (click chemistry) and the introduction of different functional groups on previous described selectors. The most recent crown-ether-based CSPs as well as their chromatographic performance presented on Table S7 (supplementary material). Some of the recent CSPs are based on calix[4]arene derivatives as chiral selectors [233,234,235]. The new derivatives are mainly prepared using click chemistry [234,235].

**Figure 6:**
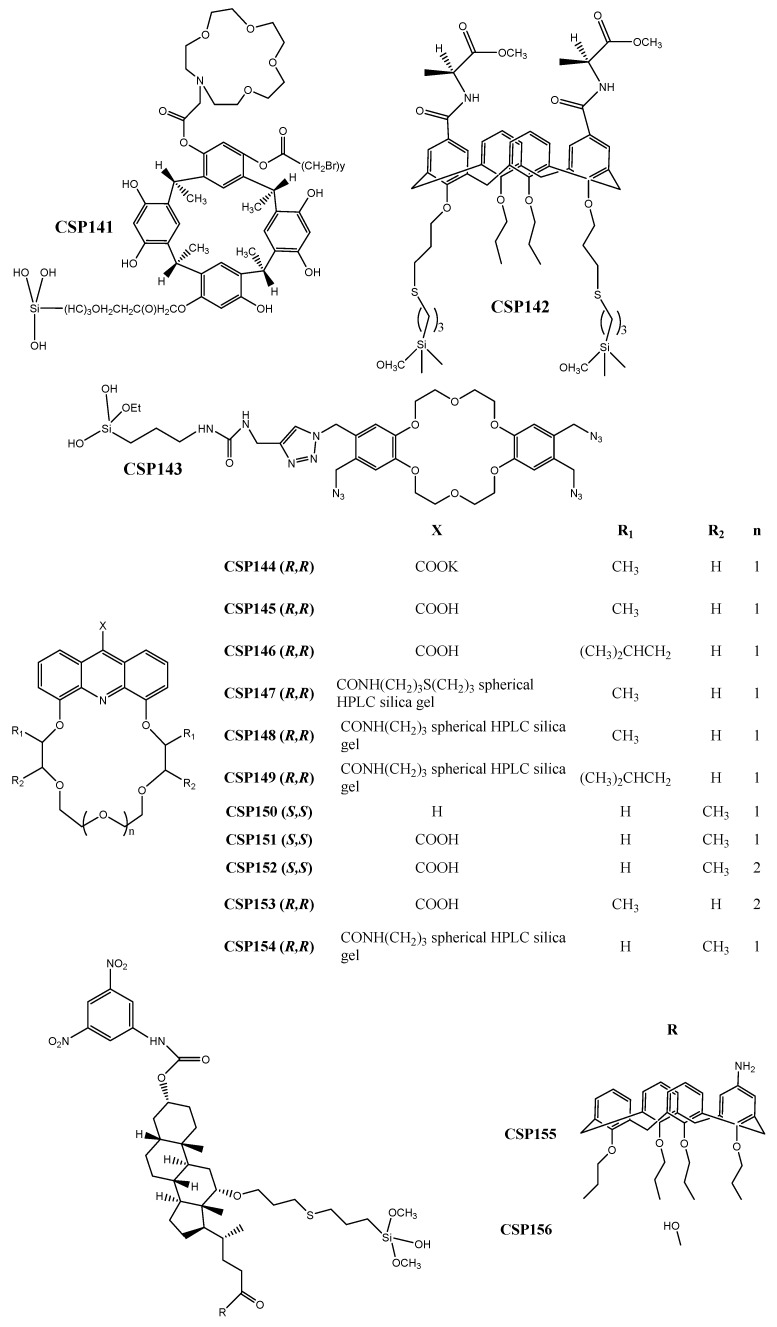
Chemical structures of crown-ether-based CSP141–156.

Accordingly, the CSP141 (Figure 6) comprising the aza-15-crown-5-capped methylcalix[4]resorcinarene derivative was developed by Ma et al. [233]. Structurally, the CSP possesses two key recognition sites, enhancing the possibility of interactions and, consequently, enantioseparation of analytes. The robustness of the CSP was highlighted since it could operate on different elution modes with a short analysis time (for example, k_1_ = 0.08 for *m*-nitrophenol).

Yaghoubnejad et al. [234] prepared a calix[4]arene functionalized with two l-alanine units to develop CSP142 (Figure 6), through covalent binding between the allyl groups at the lower rim of the chiral selector and the chromatographic support, using click chemistry. The CSP142 was able to enantioseparate both π-acidic and π-basic analytes. It was suggested that the used technique could easily be adapted to other derivatives to obtain improved CSPs. The maximum resolution value achieved was 1.43 and the separation factor was 2.00 for mandelic acid.

Click chemistry has also been explored, by Li et al. [235], for the preparation of a click-dibenzo-18-crown-6-ether-based CSP (CSP143) (Figure 6). The effect of pH and concentration of salt in the mobile phase on chromatographic parameters was evaluated [235]. It was found that the retention of strong acids decreased with the increment on salt concentration. Regarding the pH values, the retention of both acidic and basic analytes decreased with its reduction. The retention factors were good with a minimum of 0.10 for uracil.

CSPs comprising carboxyl derivatives of crown ethers as chiral selectors were also prepared. Németh et al. [236] synthetized derivatives of acridino-crown ethers containing a carboxyl group to obtain eleven CSPs (CSP144–154) (Figure 6). The CSPs were developed taking into account some structural features that could favor the interactions crucial for chiral recognition mechanisms. Due to the rigidity of the tricyclic ring system, the enantioselectivity was improved [236]. The obtained performance was reasonable with a maximum resolution value of 1.20 for 1-(1-naphthyl)-ethylamine, and a separation factor of 2.05 for 1-(4-nitrophenyl)-ethylamine hydrogen chloride.

The development of hybrid crown-ether-based CSPs was also reported. Deoxycholic-calix[4]arene hybrid-type selectors were synthetized, by Yaghoubnejad et al. [237], aiming to enhance the interactions of the obtained CSPs (CSP155–156) (Figure 6). The calix[4]arene unit was fundamental for the mechanisms of chiral recognition being responsible for the establishment of hydrophobic and π-π interactions, important for inclusion complexes formation. The presence of an acidic or basic modifier in mobile phase was beneficial for enantioresolution of acidic or basic analytes [237]. Relatively to its performance, a maximum resolution value of 3.93 and a separation factor of 4.30 were obtained for mandelic acid.

### 2.8. Cyclofructan-Based CSPs

Cyclofructans are the most recent type of CSPs being introduced, in 2009, by Armstrong et al. [238]. Moreover, they demonstrated that suitable derivatized of cyclofructans presented a superior enantioselectivity in comparison with native cyclofructans [238].

Cyclofructans are cyclic oligosaccharides formed by units of D-fructofuranose β(2→1) linked together [45]. They are also described as a crown-ether nucleus rounded by fructofuranose units, with its number between 6 and 8 [239]. Each unit has four stereogenic centres [88]. In opposition to cyclodextrins, the interior of the nucleus is hydrophilic [45]. The mechanism of chiral recognition is based on the formation of a complex, which is driven by polar interactions, including dipole-dipole and hydrogen-bond interactions [45]. Therefore, the analytes to enantioseparated should not be hydrophobic and may have hydrogen-acceptor and polarizable groups next to stereogenic center [240]. The acidic hydrogen-bond play an important role on chiral recognition, thus, the presence of a polarizable group that causes steric hindrance to the basic portion of the cyclofructan is favorable [240]. The main advantages of this type of CSPs are their high loadability and versatility, as it is able to enantioseparate basic, acidic, and neutral analytes [239]. Moreover, they can be used in different elution modes [239]. Although cyclofructan-based CSPs are recent, some reviews can be found related to its developments and applications [45,55,85,87,88,90]. The elucidation of their chiral recognition mechanisms has been the focus of some studies to clarify the interactions between the CSP and the analytes [45]. The most recent cyclofructan-based CSPs and its chromatographic behavior are presented in Table S8 (supplementary material). The latest developments include the synthesis of new derivatives of cyclofructan as chiral selectors, the preparation of new chromatographic supports, and the application of different immobilization strategies.

**Figure 7:**
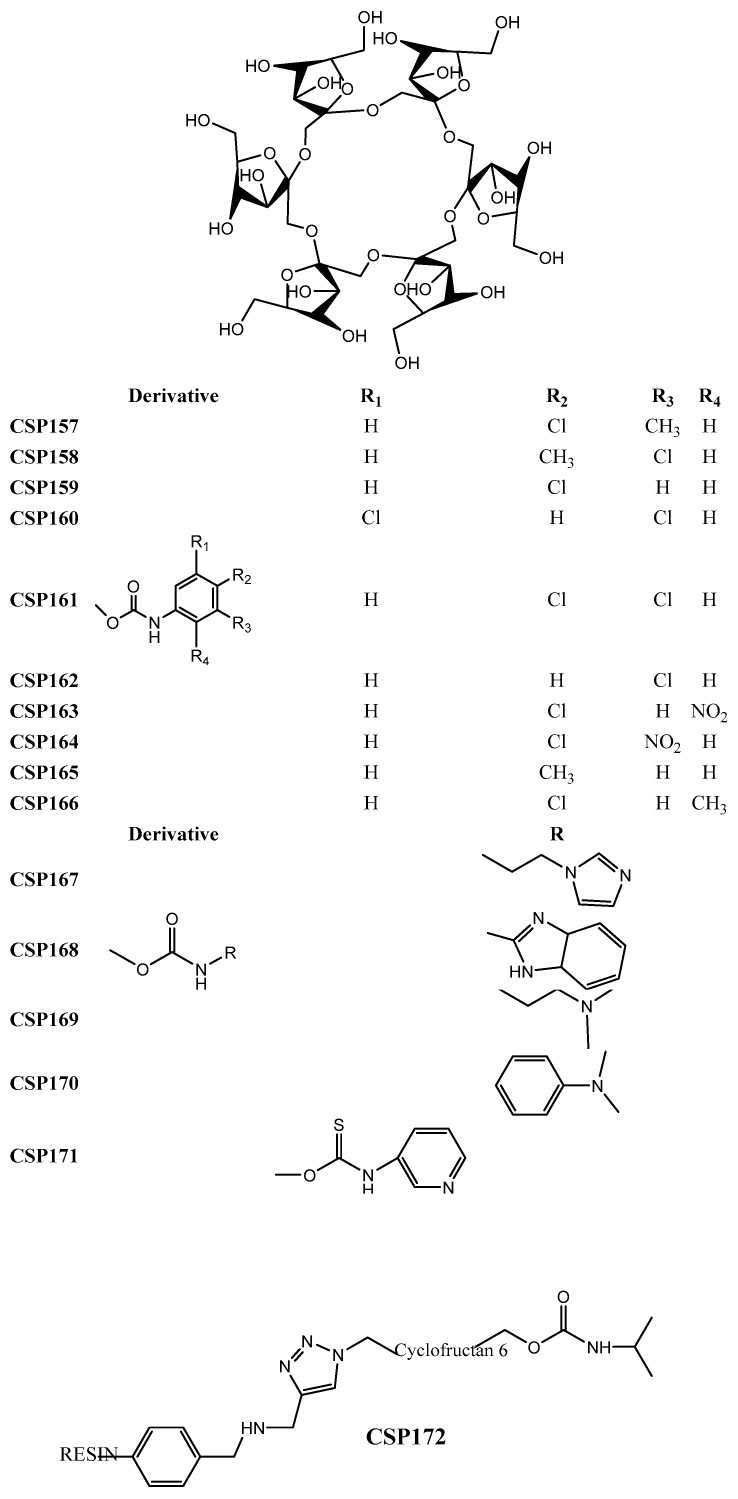
Chemical structures of cyclofructan-based CSP157–172.

In order to evaluate the effect of electron-donating and electron-withdrawing groups on enantioselectivity, Khan et al. [239] synthetized chlorinated aromatic derivatives of cyclofructan 6 and developed CSP157–166 (Figure 7). The presence of a chlorine proved to be beneficial for enantioselectivity, in opposition to nitro group, especially in the ortho position of the aromatic ring, which negatively affected the chiral recognition [239]. A maximum resolution value of 6.90 for 2-2′-binaphthol, and a separation factor of 2.05 for Tröger’s base were obtained.

The influence of the degree of substitution, as well as the size of the substituents, was researched by Padivitage et al. [241] by preparing CSP167–171 (Figure 7), with basic derivatives cyclofructan 6 as a selector. It was concluded that bulky groups caused steric hindrance and that a high degree of substitution (up to six substituents) negatively affected the enantioselectivity. Moreover, it was found that charged cyclofructans did not possess a superior ability of enantiorecognition [241]. Relatively to the performance of the CSPs, as an example, separation factor and resolution values of 1.43 and 3.10 were obtained for warfarin.

An alternative technique for preparation of a CSP was presented by Qiu et al. [242] (CSP172) (Figure 7), using click chemistry to immobilize the chiral selector, cyclofructan 6, to a resin. The resin was chosen as chromatographic support due the advantages inherent to this material, such as high adsorption capacity, high mechanical strength, lower cost, and reduced sensitivity to pH. Although the chromatographic results were only reasonable, with a resolution of 1.40 for *trans*-1-amino-2-indanol, and a separation factor of 1.41 for *N*-*p*-tosyl-1,2-diphenylethylenediamine, the stability and reproducibility of the CSP were emphasized [242].

Similarly to other types of CSPs, the applications of cyclofructan-based CSPs are becoming more prominent [243,244].

### 2.9. Molecularly-Imprinted CSPs

A different approach to chiral separation has been applied by using molecularly-imprinted CSPs. The synthesis of artificial selectors that are specific for a selected target (template) [245] is the principle of this type of CSPs. Each molecular imprinted CSP can only be applied for a specific type of analytes, as they are frequently applied on preparative enantioseparation and extraction of the desired compounds [246].

Several reviews have been devoted to molecularly-imprinted CSPs, mainly focusing on their developments and the different fields of application [45,88,246,247,248,249,250,251,252,253]. Although the developments concerning this type of CSPs are becoming more usual, their enantioresolution performance is currently not competitive in comparison to the existing CSPs. The most recent developments comprised the adaptation of this type of CSP to monoliths, nanoparticles, and predominantly to polymers. The introduction of different supports such as alginate microspheres [254] or polymer functionalized with quantum dots [255] were also reported as well as the description of different functional monomers and crosslinking agents [256,257,258,259].

Recently, Gutierrez-Climente et al. [245] prepared a new CSP by molecularly-imprinted nanoparticles on silica beads to reduce the tailing effect, commonly observed with this type of CSPs, through the reduction of particle size. The influence of some factors, such as the buffer percentage and concentration, pH, temperature, and column length, on chromatographic performance was evaluated. Regarding the chromatographic results, a maximum resolution value of 1.44 and a separation factor of 1.45 were obtained for citalopram.

In another study, Yang et al. [260] prepared a molecularly-imprinted polymer on porous silica gel microspheres to improve the chromatographic performance of a previous developed CSP and to reduce the analysis time. The new CSP demonstrated a higher affinity than the nonimprinted polymer with the silica gel, and selectivity for the target analyte, oseltamivir, with a retention factor of 13.5.

The optimization of the capacity of enantioseparation of a molecularly-imprinted monolith using a molecular crowding agent was recently reported by Wang et al. [261]. The main aim was to enhance the interactions between the CSP and the target analyte (*S*)-amlodipine. The composition of mobile phase, ionic strength, pH, and content of organic modifier were also taken into account when attempting to improve chromatographic performance [261].

### 2.10. Other CSPs

Despite the CSPs already mentioned, there are other types of CSPs, such as ligand-exchange, based on synthetic polymers, among others. The ligand-exchange CSPs do not present significant recent developments. Regarding polymers, several synthetic polymers can be used as selectors of CSPs [262]. Nevertheless, despite the interest in this type of material, synthetic polymer-based CSPs are not yet commercialized.

Regarding synthetic polymers, their classification can be based on the type of polymerization, as addition or condensation polymers and cross-linked gels, which are prepared resorting to molecular imprinted technique [263]. A synthetic and optically active polymer can be used for preparation of CSPs if it possesses a helical conformation, which contributes to the wide range of applications and effective separations [264]. The chiral recognition mechanism is based on hydrogen-bond, π-π interactions, and steric factors [45]. 

As for the other types of CSPs, the CSPs comprising synthetic polymers were the focus of several reviews [45,55,262,263,264,265,266,267]. The most recent developments are related with the introduction of monoliths [267], specifically of nanoparticles and hybrid monoliths. Ding et al. [264] also reported the use of smart polymers.

Recently, Maeda et al. [268] synthetized derivatives of optically active poly(diphenylacetylene) with chiral and achiral substituents. The helical structure of the polymer demonstrated to influence the enantioselectivity. The performance of the CSP was promising, affording excellent enantioselectivity and resolution, with α = 19.3 and Rs = 15.7 for ruthenium (III) acetylacetonate.

In another recent study, optically active π-conjugated polymers formed by alternated units of thieno[3–b]cthiophene and glucose-linked biphenyl were prepared, with its backbone conformation important for the enantioseparation of the obtained CSP [269]. Its performance was satisfactory with a maximum separation factor of 1.56 for cobalt (II) acetylacetonate.

A stable, porous, and crystalline organic polymer was introduced by Zhang et al. [270] highlighting its enhanced stability and resolution. The enantioresolution performance of the obtained CSP was reasonable with a separation factor value of 1.21 for *trans*-metoconazole, and a resolution of 2.56 value for *p-*nitrochlorobenzene.

Additionally, it is important to highlight the introduction of chip-based columns, motivated by the same factors than the transition for UHPLC, i.e., reduction of analysis time and improve the efficiency; the adaptation of LC remains challenging due to technical aspects [271,272,273]. An advantage focused for this type of columns was the simplicity of the process of its production as referred in the first report by Manz et al. [274]. More recently, they have been applied to extraction methodologies [275,276]. Despite the incorporation of micro and nanoparticles, this remains a challenging issue [273]; the introduction of monolith chip-based columns has already been reported [277].

## 3. Conclusions

The development of new CSPs for LC is a continuous and challenger issue covering various types of CSPs. This review gathered the most recent developments associated to different types of CSPs providing an overview of the advances that are occurring on this research area.

The most recent strategies, summarized in Figure 1, comprised the introduction of new chiral selectors or new chromatographic supports, and the application of different immobilization or coating methodologies for preparation of the CSPs. Regarding the chiral selectors, novel structures or analogues related to previously reported selectors were described as well as the use of hybrid selectors. The focus in chromatographic supports with lower particle size, the innovation related to the application of new materials such as monoliths and core-shell particles, as well as the use of hybrid supports, were also reported. In addition, several non-conventional approaches for immobilization or coating the chiral selectors to the chromatographic support were included, with particular emphasis to click chemistry as well as new encapsulation techniques or thermal immobilization without the use of chemical reagents, among others. Regardless of the type of CSPs, the main objectives of the development strategies were similar, concerning the improvement of the enantioresolution performance of the CSPs, as well as the increase of versatility and range of applications. Additionally, the transition to UHPLC and the possibility of the new CSPs to be used in all elution modes or using mobile phases compatible with mass spectrometric detection has also been underscored.

Even though several innovative strategies have been applied and several new CSPs were recently developed, they do not yet go beyond the exploratory stage. Nevertheless, the various strategies presented constitute an important trigger aiming to achieve new CSPs, as commercially viable products with high versatility and broad range of analytical and preparative applications or, on the contrary, as exceptionally efficient for the enantioresolution of specific target analytes.

In our opinion, the development of efficient chromatographic tools for LC enantioresolution is a subject that should continue to receive special attention, since it has constructive repercussions in several other research areas, such as biomedical, toxicology and forensic sciences, environment, food and fragrances, industry, among others. Moreover, the goal of developing a universal CSP remains a dream to be reached by those working in this research area.

## Supplementary Materials

The following are available online at https://www.mdpi.com/1420-3049/24/5/865/s1, Table S1: Recent developments of polysaccharide-based CSPs, Table S2: Recent developments of protein-based CSPs, Table S3: Recent developments of cyclodextrin-based CSPs, Table S4: Recent developments of macrocyclic-based CSPs, Table S5: Recent developments of donor-acceptor or Pirkle-type CSPs, Table S6: Recent developments of ion-exchange-based CSPs, Table S7: Recent developments of crown-ether-based CSPs, Table S8: Recent developments of cyclofructan-based CSPs.
