# Supplementary material for: Chiral Stationary Phases for Liquid Chromatography: Recent Developments
Source: Molecules. 2019 Feb 28;24(5):865. doi: 10.3390/molecules24050865 (PMC6429359; doi:10.3390/molecules24050865)
Supplement: Supplementary file 1 [file molecules-24-00865-s001.pdf]

Supplementary Material

Chiral stationary phases for liquid chromatography: recent developments

Joana Teixeira[1], Maria Elizabeth Tiritan[1,2,3], Madalena M. M. Pinto[1,2], Carla Fernandes[1,2*]

[1] Laboratório de Química Orgânica e Farmacêutica, Departamento de Ciências Químicas, Faculdade de Farmácia, Universidade do Porto, Rua de Jorge Viterbo Ferreira, 228, 4050-313 Porto, Portugal; jbteixeira@live.com.pt (J.T.); elizabeth.tiritan@iucs.cespu.pt (M.E.T.); madalena@ff.up.pt (M.M.M.P.)

[2] Interdisciplinary Centre of Marine and Environmental Research (CIIMAR), Edifício do Terminal de Cruzeiros do Porto de Leixões, Av. General Norton de Matos s/n, 4050-208 Matosinhos, Portugal

[3] Cooperativa de Ensino Superior, Politécnico e Universitário (CESPU), Instituto de Investigação e Formação Avançada em Ciências e Tecnologias da Saúde (IINFACTS), Rua Central de Gandra, 1317, 4585-116 Gandra PRD, Portugal

* Correspondence: cfernandes@ff.up.pt (C.F.); Tel.: +351-22-042-8688

Table S1. Recent developments of polysaccharide-based CSPs.

CSP	Analytes	Mobile phase (% *v/v*)		Separation factor (α)	Resolution factor (Rs)	Retention factor (k1)	Reference
**CSP1-2**	1-(9- Anthryl)-2,2,2-trifluoroethanol, *trans*-stilbene oxide, Tröger's base, benzoin, 2-phenylcyclohexanone and flavanone	HEX:IPA (90:10)		1.11-1.64	1.67-7.60	1.08-8.14	[94]
		HEX:$CHCl_3$:IPA (90:10:1)		1.12-1.84	2.17-9.59	1.31-9.21	
		HEX:THF:IPA (90:10:1)		1.08-1.44	1.19-6.27	1.37-11.3	
**CSP3-27**	Aromatic and cyclic compounds	HEX:IPA (90:10)		1.02-2.87	-	0.22-26.6	[100]
**CSP28-35**	Tröger base; 2-phenylchroman-4-one; 1-(2-naphthyl)-ethanol; methyl phenyl sulfoxide; 1-phenylethanol; mephobarbital; 4-phenyloxazolidin-2-one; 1-(1-phenylethyl)-3-(*p*-tolyl)urea; 1-(2,4-dichlorophenyl)-2-(1*H*-imidazol-1-yl)ethanol; benzoin; 1-(1-(4-methoxyphenyl)ethyl)-3-phenylurea; aminoglutethimide; glutethimide; citalopram hydrobromide; efavirenz; *N*-(1-(4-methoxyphenyl)ethyl)-3,5-dinitrobenzamide; voriconazole; 4-(4-(dimethylamino)-1-(4-fluorophenyl)-1-hydroxybutyl)-3-(hydroxymethyl)benzonitrile	HEX:IPA (90:10)		1.04-3.76	0.17-10.3	0.38-26.1	[97]
		HEX:EtOH (90:10)		1.02-6.72	0.25-18.1	0.34-32.5	
		HEX:EtOH:MeOH (90:5:5)		1.06-2.69	0.24-16.2	0.32-23.5	
**CSP36-42**	Tröger base; 2-phenylchroman-4-one; 1-(2-naphthyl)-ethanol; methyl phenyl sulfoxide; 1-phenylethanol; mephobarbital; 4-phenyloxazolidin-2-one; 1-(1-phenylethyl)-3-(*p*-tolyl)urea; 1-(2,4-dichlorophenyl)-2-(1*H*-imidazol-1-yl)ethanol; 4-methyl-*N*-(1-phenylethyl)benzamide; benzoin; 1-(1-(4-methoxyphenyl)ethyl)-3-phenylurea; aminoglutethimide; citalopram hydrobromide; efavirenz; *N*-(1-(4-methoxyphenyl)ethyl)-3,5-dinitrobenzamide; omeprazole sodium; voriconazole; 4-(4-(dimethylamino)-1-(4-fluorophenyl)-1-hydroxybutyl)-3-(hydroxymethyl)benzonitrile	HEX:IPA (90:10)		1.03-3.16	0.21-14.1	2.49-33.2	[95]
		HEX:EtOH (90:10)		1.04-8.64	0.23-11.6	1.70-36.5	
		HEX:EtOH:MeOH (90:5:5)		1.02-5.38	0.22-10.0	1.48-21.7	
**CSP43-48**		HEX:IPA (90:10)		1.04-4.32	0.10-8.05	0.28-47.3	[96]
		HEX:EtOH (90:10)		1.03-2.42	0.17-7.75	0.24-28.0	
		HEX:EtOH:MeOH (90:5:5)		1.05-3.12	0.14-11.9	0.28-20.0	
**CSP49-51**	Taladafil	HEX:EtOH	80:20	1.42-2.15	2.08-4.72	2.87-12.7	[102]
			90:10	1.07-1.81	0.10-2.55	1.86-37.9	
**CSP52-55**	*trans*-Stilbene oxide; 2,2-dihydroxy-6,6-dimethylbiphenyl;	HEX:IPA (90:10)		1.04-3.06	0.24-11.7	0.43-25.6	[101]

	benzoin; 2-phenyl-cyclohexanone; tröger base; 1-(9-anthryl)-2,2,2-tri-fluoroethanol; cobalt(III) *tris*(acetylacetonate); 1,2,2,2-tetraphenylethanol; flavanone; *trans*-cyclopropanedicarboxylic acid dianilide	HEX:EtOH (90:10:1)		1.04-2.31	0.10-10.6	0.31-23.9	
		HEX:EtOH:MeOH (90:5:5)		1.03-1.98	0.19-9.03	0.32-21.1	
		HEX:$CHCl_3$:IPA (90:10:1)		1.12-1.84	2.17-9.59	1.31-9.21	
		HEX:THF:IPA (90:10:1)		1.08-1.44	1.19-6.27	1.37-11.3	
**CSP56**	Tröger base; 2-phenylchroman-4-one; 1-(2-naphthyl)-ethanol; methyl phenyl sulfoxide; 1-phenylethanol; mephobarbital; 4-phenyloxazolidin-2-one; 1-(1-phenylethyl)-3-(*p*-tolyl)urea; 1-(2,4-dichlorophenyl)-2-(1*H*-imidazol-1-yl)ethanol; 4-methyl-*N*-(1-phenylethyl)benzamide; benzoin; 1-(1-(4-methoxyphenyl)ethyl)-3-phenylurea; aminoglutethimide; citalopram hydrobromide; efavirenz; *N*-(1-(4-methoxyphenyl)ethyl)-3,5-dinitrobenzamide; omeprazole sodium; voriconazole; 4-(4-(dimethylamino)-1-(4-fluorophenyl)-1-hydroxybutyl)-3-(hydroxymethyl)benzonitrile	HEX:IPA (90:10)		1.06-4.32	0.03-6.75	0.25-15.0	[98]
		HEX:EtOH (90:10)		1.07-3.59	0.11-8.49	0.17-4.96	
**CSP57**	β-blockers, anti-inflammatory drugs, norepinephrine-dopamine reuptake inhibitor, catecholamines, sedative hypnotics, anti-histaminics, anticancer drugs, antiarrhythmic drug, flavonoids, amino acids, anti-platelet agents, immunomodulatory drugs	MeOH:H_2O	30:70	1.20-2.90	0.77-2.80	-	[103]
			30:70 with 1% TFA	1.20-1.86	<1.00-1.66	-	
			40:60	1.09-3.80	<1.00-2.66	-	
			50:50	1.10-1.52	<1.00-2.00		
			80:20	1.06-1.34	<1.00-1.32	-	
			90:10	1.35-1.49	0.99-1.30	-	
		ACN:H_2O (10:90)		1.13-2.80	<1.00-2.30	-	
		HEX:IPA	50:50	1.20-1.90	<1.00-2.10	-	
			90:10	1.11-2.00	<1.00-2.40	-	
			90:10 with TFA	1.10-1.90	<1.00-2.00	-	
**CSP58**	*trans*-Stilbene oxide; benzoin; 2-phenylcyclohexanone; flavanone; benzoin; praziquantel; benzylpenicillin; cypermethrin; ketoconazole; equol; ibuprofen	HEX:IPA	98:2	1.18-8.42	1.23-1.88	0.49-1.57	[99]
			95:5	1.38-7.60	0.72-1.61	0.32-1.72	
			90:10	1.12-8.11	0.41-2.10	0.35-7.90	
			85:15	4.02-4.44	1.03-1.25	0.31-1.51	
			80:20	1.20-4.78	0.73-1.60	1.11-3.25	
			70:30	1.20-3.52	0.55-1.04	0.48-3.32	
			60:40	3.03-3.28	1.60-1.77	0.37-1.21	
			50:50	1.74-3.13	1.23-2.61	0.73-0.99	
			40:60	7.00	1.62	0.18	
**CSP59**	Etozoline, 2-benzylsulfinyl benzamide, 2-(3-	MeOH		2.10-15.3	2.0-11.0	0.09-0.27	[106]

	bromobenzylsulfinyl)-benzamide, 2-(4-methylbenzylsulfinyl)-benzamide, 2-(benzylsulfinyl)-*N*,*N*-dimethyl benzamide, 2-(benzylsulfinyl)-*N*-methyl benzamide, 2-(2-methylbenzylsulfinyl)-benzamide, 2-(3-methylbenzylsulfinyl)-benzamide						
**CSP60**	2,2,2-Trifluoro-1-(9-anthryl)ethanol; benzoin; methyl mandelate; *trans*-stilbene oxide	HEX:IPA	95:5	1.96-2.41	-	0.94-4.12	[108]
			98:2	1.27-1.49	-	9.05-10.2	
**CSP61**	Omeprazole	$MeOH:H_2O$ (40:60)		3.10	-	1.00	[110]

ACN: acetonitrile; DEA: diethylamine; HEX: *n*-hexane; EtOH: ethanol; IPA: 2-propanol; MeOH: methanol; TFA: trifluoroacetic acid; THF: tetrahydrofuran.

Table S1. Recent developments of protein-based CSPs.

CSP	Analytes	Mobile phase (% *v*/*v*)	Affinity constant (K_a; M^{-1})	Resolution factor (R_S)	Retention factor (k_1)	Reference
**CSP62-64**	Benzoin; ibuprofen; chlorpheniramine; propranolol; oxprenolol	$DiPO_4$- $DiNaPO_4$ (pH 5.1):EtOH (90:10)	-	0.59-14.2	3.42-38.6	[126]
**CSP65-66**	Carbamazepine; disopyramide; imipramine; lidocaine; *S*-propanolol	0.10 M potassium PBS (pH 5.0)	6.40×10^4-2.10×10^6	-	2.10-69.4	[124]
**CSP67**	Propranolol, alprenolol, oxprenolol, pindolol	20 mM sodium $DiPO_4$– $DiNaPO_4$ (pH 6.8):IPA (95:5)	-	0.97-10.7	0.41-34.6	[128]
**CSP68**	Warfarin, verapamil, carbamazepine	0.067 M PBS (pH 7.4)	5.30×10^3-2.60×10^5	0.61-1.39	4.20-233	[130]
**CSP69**	Disopyramide, chlorpromazine, imipramine, propranolol, warfarin	0.067 M PBS (pH 7.4)	0.26×10^4-90.0×10^6	-	0.54-14.1	[131]
**CSP70**	Ketoprofen, fenoprofen, indoprofen, ibuprofen, flurbiprofen	MeOH:potassium $DiPO_4$ (pH 4.5) (50:50)	-	0.79-2.14	1.80-17.9	[133]
**CSP71-72**	Warfarin, tryptophan	0.067 M PBS (pH 7.4)	-	1.49-4.52	1.49-121	[125]

$DiNaPO_4$: disodium hydrogen phosphate; $DiPO_4$: dihydrogen phosphate; EtOH: ethanol; IPA: 2-propanol; MeOH: methanol; PBS: phosphate buffer.

Table S2. Recent developments of cyclodextrin-based CSPs.

CSP	Analytes	Mobile phase (% *v/v*)		Separation factor (α)	Resolution factor (R_S)	Retention factor (k_1)	Reference
**CSP73**	Flavanone; 4′-hydroxyflavanone; 6-hydroxyflavanone; 1-(*p*-tolyl)but-3-en-1-ol; *trans*-1,3-diphenyl-2-propen-1-ol; 3-(4-methoxyphenyl)-5-phenyl-4,5-dihydro-1,2-oxazole; 1-(3-(*p*-tolyl)-4,5-dihdroisoxazol-5-yl)pyrrolidine-2-one; 1-(3-(3-nitrophenyl)-4,5-dihydroisoxazol-5-yl)pyrrolidine-2-one	MeOH:H_2O	60:40	1.18-2.24	0.98-4.17	1.02-7.85	[152]
	3′-Hydroxyflavanone; 6-methoxyflavanone; 7-methoxyflavanone; 1-(3-(4-chlorophenyl)-4,5-dihydroisoxazol-5-yl)pyrrolidine-2-one		70:30	1.75-2.39	3.02-4.40	1.11-7.63	
	1-(4-Chlorophenyl)ethanol; 1-(4-bromophenyl)ethanol		90:10	1.38-1.64	0.71-0.88	0.12-0.27	
	Naringenin	MeOH:5%TEAA buffer (pH 4.0) (70:30)		1.17	0.61	1.23-1.44	
**CSP74**	Flavonoids, β-blockers and isoxazolines	MeOH:H_2O	60:40	1.01-1.29	0.20-1.96	2.70-9.47	[151]
			70:30	1.03-1.70	0.25-6.03	1.26-3.71	
			80:20	1.05-1.70	0.67-5.93	1.15-5.61	
**CSP75**	Flavonoids, aromatic alcohols, acidic drugs, β-blocker, and amino acids.	MeOH		1.09-1.83	0.64-8.07	-	[159]
		MeOH:H_2O (10:90)		1.08	1.68	-	
		MeOH:TEAA (1% pH 4.0)		1.06-2.24	0.76-4.32	-	
		MeOH:TEAA (1% pH 5.0)		1.08	1.06	-	
		HEX:EtOH	60:40	1.19-2.37	1.90-5.09	-	
			70:30	1.22-2.55	2.10-7.32	-	
			80:20	1.26-2.71	2.35-7.84	-	
			90:10	1.34-2.60	3.7.3-8.50	-	
		HEX:IPA	60:40	1.23-2.71	2.27-5.09	-	
			70:30	1.26-2.75	2.44-5.08	-	
			80:20	1.32-2.76	2.69-5.29	-	
			90:10	1.37-2.73	2.91-5.27	-	
		HEX:MeOH:EtOH	90:5:5	1.36-2.12	5.46-9.84	-	
			80:10:10	1.44-2.41	3.85-8.85	-	
			70:15:15	1.40-2.26	3.34-8.22	-	
			60:20:20	1.37-2.14	3.02-7.95	-	

		HEX:MeOH:IPA	90:5:5	1.42-2.13	5.35-8.52	-	
			80:10:10	1.45-2.46	3.56-8.06	-	
			70:15:15	1.42-2.29	3.04-7.48	-	
			60:20:20	1.38-2.14	2.71-7.37	-	
**CSP76**	Carvedilol	ACN:MeOH:AcOH:TEA	(99:1:2:1.8)	1.30	1.97	8.20-10.7	[149]
	Atenolol		(90:10:1:0.9)	1.15	1.62	9.60-11.1	
	Metoprolol		(95:5:0.5:0.4)	1.17	1.67	7.31-8.58	
	Esmolol		(95:5:0.4:0.4)	1.18	1.69	6.28-7.38	
	Propranolol		(95:5:0.5:0.5)	1.16	1.48	6.79-7.87	
	Arotinolol; carteolol; bisoprolol		(90:10:1:1)	1.07-1.14	0.52-1.45	4.70-8.40	
	Pindolol		(99:1:1:0.8)	1.05	<0.50	5.36-5.63	
**CSP77-80**	Neutral, basic, and acidic analytes	0.50% NH_4Ac:MeOH	100:0	1.14-2.90	1.98-7.01	1.37-10.41	[160]
			95:5	1.19	1.96	7.81	
			90:10	5.97	9.56	0.42	
			80:20	1.10-1.35	0.67-3.98	1.98-7.49	
			70:30	1.17-1.25	1.13-1.75	1.19-4.14	
			60:40	1.10	0.95	8.72	
			20:80	6.08	6.70	0.19	
**CSP81-85**	Nine Cyclic and aromatic compounds	HEX:IPA (90:10)		1.13-2.87	-	0.25-5.47	[148]
**CSP86-88**	β-Nitroethanol and derivatives, aromatic alcohols, amino acids derivatives, and chiral drugs	ACN:MeOH:AcOH:TEA	480:20:0.5:1	1.01-5.18	0.39-22.0	0.39-9.99	[150]
			480:20:1:1	1.04-15.5	0.50-21.2	0.37-8.03	
			480:20:1:0.5	1.05-3.36	0.55-12.4	1.44-6.77	
		MeOH:H_2O (50:50)		1.07-2.06	0.72-5.78	1.60-14.0	
**CSP89-92**	Isoxazoline derivatives, flavonoids, dansyl amino acids, styrene oxide, and Tröger's base	MeOH:H_2O	30:70	1.05-3.30	1.00-7.15	0.39-12.2	[161]
			60:40	1.12	3.55	8.12-9.08	
			80:20	1.11-1.21	2.46-3.05	1.60-3.84	
			70:30	1.05-2.04	0.94-7.06	0.69-9.84	
			25:75	1.08-1.12	2.01-3.11	2.70-3.86	
		MeOH:1% TEAA buffer (pH 4.9) (70:30)		1.04-1.33	0.04-3.36	0.95-7.51	
		ACN:H_2O	15:85	1.03-1.08	0.82-1.82	1.49-5.93	
			30:70	1.05-2.28	1.13-3.30	0.15-11.6	
			10:90	1.01-1.03	0.19-1.05	7.54-11.8	

			35:65	1.04-1.17	1.39-5.55	2.34-13.3	
			50:50	1.05-1.71	1.07-12.7	1.21-3.19	
			40:60	1.05-1.15	1.20-1.90	1.15-3.35	
**CSP93**	1-Phenylethanol, 1-phenyl-2-propanol, mandelonitrile, diclofop, 1-(4-hlorophenyl)-ethanol, 1,2,3,4-tetrahydro-naphthalen-1-ol, 1,3-diphenylpropane-1,3-diol, triadimenol, albendazole sulfoxide, trazodone, metalaxyl, promethazine, 1-(2-methoxyphenyl)-ethanol, 1,3-diphenylprop- 2-en-1-ol, chlorpheniramine, propranolol, metoprolol, atenolol, mexiletine	HEX:IPA:TFA:DEA	90:10:0:0	1.14-1.63	0.58-4.65	0.24-5.75	[154]
			70:30:0:0	1.11-1.18	0.69-0.71	1.20-2.37	
			90:10:0.1:0	1.10-1.17	0.60-1.27	4.86-6.01	
			95:5:0:0.1	1.14	1.87	7.23-8.25	
		MeOH:1%TEAA (pH 4.0) (40:60)		1.19-1.38	0.97-3.70	0.97-2.65	
		MeOH:0.2% FA (35:65)		1.12	0.72	8.91-9.99	
		MeOH:H_2O (50:50)		1.16	3.73	4.13-4.79	
		ACN:0.1% TEAA (pH 5.2) (15:85)		1.15-1.39	0.65-3.10	0.66-2.91	
**CSP94-97**	Pyrrolidine compounds	ACN:MeOH:AcOH:TEA	480:20:0.5:1	1.04-2.99	0.54-6.43	0.71-3.95	[155]
			480:20:1:1	1.02-1.82	0.45-3.78	1.13-3.32	
			480:20:1:0.5	1.05-3.65	0.57-3.51	0.62-1.52	
		HEX:IPA	50:50	1.02-2.10	0.74-5.22	1.29-2.61	
			75:25	1.08-1.41	0.68-5.14	1.37-2.67	
			90:10	1.04-2.84	2.00-9.31	1.43-3.70	
**CSP98**	Alprenolol, atenolol, arterenol, desmethylcizolirtine, indoprofen, glafenine, etodolac, carprofen, celiprolol, flavanone, etodolac, bufuralol, hexaconazole, pentobarbital, chlorpheniramine	MeOH:H_2O	65:35	1.07-1.11	1.14-1.41	-	[156]
			70:30	1.08	1.92	-	
			75:25	1.07	1.06	-	
			80:20	1.08-1.42	1.14-1.91	-	
			90:10	1.29-1.30	2.51	-	
**CSP99**	Benzoin, tyrosine, equol, ibuprofen, propranolol, praziquantel, 1-phenylethanol	MeOH:ACN:H_2O	30:30:40	2.10	1.87	20.9	[157]
			40:40:20	1.81	1.29	2.92	
			25:25:50	2.44	2.17	3.19	
		ACN:IPA (60:40)		38.8	1.59	1.94	
		MeOH:ACN:H_2O (0.1% ammonium acetate) (40:40:20)		2.64	1.29	7.20	
		MeOH:H_2O (0.1% ammonium acetate)	56:44	1.80	0.93	1.01	
			64:36	2.16	1.56	1.08	
**CSP100**	Promethazine, benzoin, chlortrimeton	ACN:H_2O (70:30)		-	-	1.59-2.50	[158]
		MeOH		-	-	1.13-2.68	

			-	-	3.84	3.84
	TEAA:ACN		-	-	5.56	5.56
			-	-	8.04	8.04

ACN: acetonitrile; AcOH: acetic acid; DCM: dichloromethane; DEA: diethylamine; FA: formic acid; MeOH: methanol; TEA: triethylamine; TEAA: Triethylammonium acetate; TFA: trifluoroacetic acid.

Table S4. Recent developments of macrocyclic-based CSPs.

CSP	Analytes	Mobile phase (% *v*/*v*)		Separation factor (α)	Resolution factor (R_S)	Retention factor (k_1)	Reference
**CSP101**	Norleucine, alanine, valine, methionine, leucine, norvaline, threonine, serine, phenylalanine, tryptophan	EtOH:H_2O	80:20	1.10-7.00	0.40-5.00	0.34-2.55	[171]
			90:10	1.20-9.40	0.60-5.60	0.76-6.22	
		MeOH:H_2O	90:10	1.20-4.40	0.60-5.00	0.32-1.46	
			50:50	2.33	0.67	0.20-0.46	
**CSP102**	Alanine, serine, glutamine, leucine, methionine, phenylalanine, tryptophan, haloxyfop, mandelic acid, ketorolac, sulfoxide 4, phosphine oxide	MeOH:H_2O(85:15) + 20 mM AmAc		1.07-3.45	1.15-10.7	2.74-22.5	[172]
		ACN:H_2O (85:15) + 15 mM AmAc		1.18-2.83	1.28-9.90	1.06-8.96	
		ACN:MeOH (60:40) + 0.055% AcOH + 0.03% TEA		1.20-2.45	2.29-9.83	10.7-64.2	
		A: HEX:EtOH (95:5) B: HEX:EtOH:MeOH (50:45:5)		1.06-1.41	0.86-6.02	1.47-8.62	
**CSP103-106**	Herbicides and non-steroidal anti-inflammatory drugs	500 mM AmAc buffer (pH 4.5):H_2O:MeOH (5:10:85)		1.69-2.69	2.26-3.36	0.28-1.22	[173]
		500 mM AmAc buffer (pH 4.5):H_2O:ACN (1:9:90)		1.20-1.66	1.27-2.85	0.33-3.14	
**CSP107**	Carteolol, salbutamol, clenbuterol, propanolol, acebutolol, pindolol, tertaolol, sotalol	MeOH:ACN:TEA:AcOH (85:15:0.08:0.02)		1.15-1.23	1.26-1.47	0.66-2.01	[174]
**CSP108**	50 Amino acids, pesticides, stimulants, and pharmaceuticals	MeOH:NH_4 formate	100:0.1	1.04-1.57	0.30-2.70	0.30-0.90	[175]
			30:70 (pH 3.6 16 mM)	1.05-1.50	0.60-1.90	0.40-2.50	
		ACN:MeOH:AcOH:TEA (60:40:0.3:0.2)		1.07-1.08	0.6	1.00-1.20	
		HEX:EtOH:TFA:TEA (70:30:0.3:0.2)		1.01-1.14	0.20-2.10	0.50-5.00	
**CSP109**	Benzoin	HEX:IPA	80:20	3.56-4.23	1.51-3.08	0.24-1.10	[176]
			50:50	2.33	0.67	0.20-0.46	

ACN: acetonitrile; AcOH: acetic acid; AmAc: ammonium acetate; EtOH: ethanol; HEX: *n*-hexane; IPA: 2-propanol; MeOH: methanol; NH_4: ammonium; TEA: triethylamine.

Table S5. Recent developments of donor-acceptor or Pirkle-type CSPs.

CSP	Analytes	Mobile phase (% *v*/*v*)		Separation factor (α)	Resolution factor (R_S)	Retention factor (k_1)	Reference
**CSP110**	4-Hydroxyquinoline, quinoline, 4-methylquinoline, 4-nitroquinoline, phloroglucinol, resorcinol, phenol, 2-cresol, 3-nitrophenol, metronidazole, ronidazole, tinidazole, ornidazole, ipronidazole, 3-nitroaniline, 4-nitroaniline, 2-nitroaniline, carvedilol	MeOH:H_2O	60:40	1.91-2.08	2.08-3.47	1.24-7.70	[196]
			50:50	1.63-2.43	0.91-4.13	0.78-9.65	
			40:60	1.21-1.72	0.93-2.05	1.84-7.38	
		HEX:IPA (90:10)		1.47	1.75	1.20-1.76	
**CSP111**	Mandelic acid and 2-phenylpropionic acid	0.2 M PBS		2.08-2.75	2.10-3.85	0.71-6.00	[197]
**CSP112-115**	1,1′-bi-2-Naphthol and benzoin	HEX:IPA (90:10)		1.14-9.80	0.30-2.89	0.10-34.3	[198]
**CSP116-119**	π-Acidic, π-basic, aromatic, and oxazolidinone compounds	HEX:IPA (90:10)		1.03-2.58	-	0.63-12.3	[199]
		HEX:IPA:TFA (90:10:0.1)		1.05-1.71	-	2.47-8.59	
**CSP120**	1,1′-Binaphthol, 3,5-dinitro–*N*-(1-phenylethyl) benzamide, 5-methoxy flavanone, 6-methoxy flavanone, thalidomide, 2,2,2-trifluoro-1-(9-anthryl)-ethanol	HEX:EtOH:TFA	99:1:0.1	1.03-1.12	-	0.69-9.09	[200]
			97:3:0.1	1.12	-	7.18	
			90:10:0.1	1.05	-	19.9	

ACN: acetonitrile; AcOH: acetic acid; AmAc: ammonium acetate; HEX: *n*-hexane; IPA: 2-propanol; MeOH: methanol; PBS: phosphate buffer; TFA: trifluoroacetic acid.

Table S3. Recent developments of ion-exchange-based CSPs.

CSP	Analytes	Mobile phase (% *v*/*v*)		Separation factor (α)	Resolution factor (R_S)	Retention factor (k_1)	Reference
**CSP121-125**	Alanine, arginine, asparagine, aspartic acid, cysteine, glutamine, glutamic acid, histidine, isoleucine, leucine, lysine, methionine, phenylalanine, proline, serine, threonine, tryptophan, tyrosine, valine	20 mM AmAc (pH 6.0) in H_2O:MeOH	85:15	1.39	5.04	24.9-34.7	[214]
			75:25	1.51-4.56	2.17-11.8	3.00-20.8	
			70:30	2.86	9.66	5.70-16.3	
			60:40	1.12-3.70	0.39-11.3	2.00-27.1	

**CSP126-131**	Acidic analytes, amino acids, and anti-inflammatory profens	MeOH:0.1 M AmAc (80:20) (pH = 6.0)		1.02-17.0	-	1.22-61.3	[218]
**CSP132**	*N*-protected amino acids, *α*-aryloxy carboxylic acids, non-steroidal anti-inflammatory profens, aryl amides, esterified DNB-amino acids, benzodiazepines, and binaphthol	MeOH:AcOH (98:2) + 0.5 g/100 mL AmAc		1.04-1.24	0.79-2.91	0.50-3.74	[216]
		MeOH:ACN (90:10) + 0.1% (*v*/*v*) FA + 10 mM AmFm		1.05-1.25	1.02-3.22	0.86-8.15	
		MeOH:ACN (50:50) + 0.2% (*v*/*v*) FA + 10 mM AmFm		1.06-1.26	0.85-3.19	2.31-9.43	
		HEX:DCM (75:25) + 2% (*v*/*v*) MeOH		1.08-1.49	0.93-7.33	1.28-12.1	
		HEX:DCM (50:50) + 2% (*v*/*v*) MeOH		1.18-2.06	1.63-11.0	0.81-14.8	
**CSP133**	*N*-acetyl-phenylalanine, *N*-carbobenzoxyphenylalanine, *N*-[(9*H*-fluoren-9-ylmethoxy)-carbonyl]-phenylalanine, and dichlorprop	MeOH:AcOH:AmAc	98:2:0.5	1.21-1.51	1.73-3.99	0.58-2.09	[219]
			99.7:0.3:0.07	1.22-1.54	1.81-5.20	0.95-3.50	
			99.6:0.4:0.1	1.48	4.14	1.19	
**CSP134-137**	*N*-Acetyl-phenylalanine, *N*-[(9*H*-Fluoren-9-ylmethoxy)carbonyl]phenylalanine, 2-(2,4-dichlorphenoxy)propionic acid, *N*-carbobenzoxy-phenylalanine	MeOH:AcOH:AmAc (98:2:0.5)		1.18-1.66	0.90-6.20	0.40-7.60	[215]
		MeOH:H_2O:AcOH:AmAc (58:40:2:0.5)		1.12-1.43	1.00-5.10	1.10-400	
**CSP138**	Proteinogenic amino acids	A: MeOH:H_2O (98:2) with FA and AmFm<br>B: MeOH:H_2O (98:2)	25:75 (25 mM FA and 25 mM AmFm)	1.08-1.32	0.65-0.97	0.24-2.17	[217]
			50:50 (50 mM FA and 50 mM AmFm)	1.11-1.55	0.91-4.08	0.51-3.17	
			75:25 (75 mM FA and 75 mM AmFm)	1.08-1.48	0.63-2.99	0.47-2.65	
			100:0 (100 mM FA and 100 mM AmFm)	1.27	1.99	1.97-2.47	
			99.7:0.3:0.07	1.22-1.54	1.81-5.20	0.95-3.50	
			99.6:0.4:0.1	1.48	4.14	1.19	
**CSP139-140**	Acidic analytes	MeOH:100 mM AmAc (pH = 6.0) (80:20)		1.08-2.38	1.00-13.4	1.20-35.8	[220]
		MeOH:100 mM AmAc (pH = 7.0) (80:20)		1.15-14.5	1.30-25.5	1.00-23.0	

ACN: acetonitrile; AcOH: acetic acid; AmAc: ammonium acetate; AmFm: ammonium formate; DCM: dichloromethane; FA: formic acid; HEX: *n*-hexane; MeOH: methanol.

Table S4. Recent developments of crown-ether-based CSPs.

CSP	Analytes	Mobile phase (% *v*/*v*)		Separation factor (α)	Resolution factor (R_S)	Retention factor (k_1)	Reference
**CSP141**	*o*-Nitroaniline, *m*-nitroaniline, *p*-nitroaniline, *o*-nitrophenol, *m*-nitrophenol, *p*-nitrophenol	MeOH:H_2O	100:0	-	-	0.08-0.24	[233]
			80:20	-	-	0.36-0.46	
			60:40	-	-	0.92-1.10	
			40:60	-	-	1.41-1.88	
			20:80	-	-	2.79-4.51	
			10:90	-	-	3.88-6.78	
			5:95	-	-	4.26-8.30	
		ACN:H_2O	100:0	-	-	0.11-0.42	
			80:20	-	-	0.17-0.21	
			60:40	-	-	0.64-0.75	
			40:60	-	-	1.23-1.53	
			20:80	-	-	2.02-2.57	
			10:90	-	-	2.65-5.03	
			5:95	-	-	3.30-6.46	
**CSP142**	3,5-Dinitrobenzoyl derivative of alanine, leucine, valine, methionine; diclofop-methyl, and mandelic acid	HEX:IPA:TFA	85:15:0.1	1.34-2.00	0.93-1.43	1.05-10.5	[234]
			99:1:0	1.25	0.73	2.05-2.58	
		H_2O:MeOH:AcOH	90:10:0.1	1.06	0.37	6.82-7.29	
			80:20:0.1	1.15	0.79	11.2-12.9	
			70:30:0.1	1.09-1.11	0.35-0.40	5.19-7.49	
		ACN:40 mM AmAc (20:80)		1.02-2.05	0.20-0.34	1.82-5.06	
**CSP143**	Uracil, aniline, acetanilide, phenol, 4-nitrophenol, biphenyl, benzoic acid, *p*-nitrophenol	ACN:aqueous FA (40:60)		-	-	0.10-4.32	[235]
		ACN:AmAc (pH=5.5) (30:70)		-	-	0.29-4.33	
**CSP144-154**	Aralkylamines and α-amino acid esters	ACN:25 mM AmAac (20:80)		1.23	1.20	3.08-4.00	[236]
		ACN:40 mM AmAc (20:80)		1.02-2.05	0.20-0.34	1.82-5.06	
**CSP155-156**	*N*-(3,5-Dinitrobenzoyl)-leucine, *N*-(3,5-dinitrobenzoyl)-valine, omeprazole, diclofop-methyl, mandelic acid, and pregabalin	HEX:IPA:TFA	90:10:0.1	1.16-1.34	0.60-1.65	4.90-7.85	[237]
			95:5:0.1	1.08-4.30	0.22-3.93	1.06-6.06	
		HEX:DCM:EtOH:DEA (85:15:3:0.5)		1.30	0.48-0.89	3.73-7.94	

	HEX:IPA (99:1)		1.34-1.55	0.91-1.76	0.81-1.33
	H_2O:MeOH:AcOH	85:15:0.1	1.11	0.38	5.20-5.80
		80:20:0.1	1.19	0.45	6.38-7.60
		95:5:0.1	1.13	0.82	11.0-12.5

ACN: acetonitrile; AcOH: acetic acid; AmAc: ammonium acetate; DCM: dichloromethane; DEA: diethylamine; EtOH: ethanol; FA: formic acid; HEX: *n*-hexane; IPA: 2-propanol; MeOH: methanol; TFA: trifluoroacetic acid.

Table S8. Recent developments of cyclofructan-based CSPs.

CSP	Analytes	Mobile phase (% *v*/*v*)		Separation factor (α)	Resolution factor (R_S)	Retention factor (k_1)	Reference
**CSP157-166**	Thalidomide, warfarin, furoin, Tröger's base, *t*-stilbene oxide, 2-2'-binaphthol, 2,2'-binaphthylamine	HEP:EtOH	70:30	1.02-1.11	0.50-1.10	4.06-11.2	[239]
			95:5	1.05-1.11	0.80-1.20	0.80-1.76	
			99.5:0.5	1.02-2.05	0.50-6.90	1.68-29.9	
**CSP167-171**	34 Acid, basic, and neutral analytes	HEP:EtOH (with 0.1% TFA)	70:30	1.05-1.17	1.00-1.60	1.30-5.21	[241]
			80:20	1.01-1.43	0.50-3.10	0.67-14.3	
			90:10	1.02-1.11	0.50-2.00	0.39-8.26	
			95:5	1.08-1.16	1.50-2.00	6.88-18.6	
		ACN:MeOH (with 0.3% AcOH and 0.2% TEA)	60:40	1.01-1.03	0.50-0.60	0.78-6.12	
			80:20	1.02-1.10	0.60-1.00	0.62-0.67	
			98:2	1.04-1.18	0.50-1.70	1.25-6.19	
**CSP172**	37 Derivatives of amines and alcohols	HEP:EtOH:TFA (70:30:0.1)		1.05-1.41	0.20-1.40	0.80-6.40	[242]
		ACN:MeOH:TEA:AcOH	65:35:0.2:0.5	1.02-1.32	0.20-0.95	0.17-4.54	
			70:30:0.2:0.5	1.02-1.24	0.20-1.40	1.88-5.94	
			75:25:0.2:0.5	1.09	0.60	3.66	
			80:20:0.2:0.5	1.22	1.00	0.25	

ACN: acetonitrile; AcOH: acetic acid; EtOH: ethanol; HEP: heptane; MeOH: methanol; TEA: triethylamine; TFA: trifluoroacetic acid.
